# Phenotypically diverse *Klebsiella pneumoniae* urinary tract infection isolates consistently cause pyelonephritis

**DOI:** 10.1128/iai.00508-26

**Published:** 2026-10-12

**Authors:** Grace E. Shepard, Zachary Mills, Drew A. Pariseau, Brooke E. Ryan, Jolie Lagger, Laura A. Mike

**Affiliations:** 1 Division of Infectious Diseases, Department of Medicine, University of Pittsburgh 199716, Pittsburgh, Pennsylvania, USA; 2 Department of Medical Microbiology and Immunology, University of Toledo 685614, Toledo, Ohio, USA; University of California Davis, Davis, California, USA

**Keywords:** *Klebsiella pneumoniae*, urinary tract infection, mucoidy, capsule, pyelonephritis, complement resistance

## Abstract

Urinary tract infections (UTIs) are a significant public health burden that impact millions of people every year and are highly prevalent among hospital-acquired infections. *Klebsiella pneumoniae* is globally the second most common cause of UTIs after uropathogenic *Escherichia coli* (UPEC). Thus far, the molecular mechanisms underlying pathogenesis are better understood in UPEC than in *K. pneumoniae*. UPEC is known to have virulence phenotypes such as fimbrial adhesion and evasion of complement-mediated killing. In other infection types, *K. pneumoniae* fitness has been associated with mucoidy and diverse capsular serotypes. To establish *K. pneumoniae* virulence phenotypes contributing to UTI, we examined how environmental cues regulate urovirulence-associated phenotypes in clinical *K. pneumoniae* UTI strains. These phenotypes included capsular polysaccharide properties, hemagglutination, serum resistance, adherence to bladder epithelial cells, and *in vivo* fitness. We found that clinical *K. pneumoniae* UTI isolates’ phenotypes are highly heterogeneous and can change in response to human urine. Despite *K. pneumoniae* clinical isolates presenting heterogeneous fitness properties, all similarly colonize the urinary tract. These results suggest that additional fitness factors contribute to *K. pneumoniae* uropathogenesis. Identifying these shared fitness factors will provide mechanistic insights into *Klebsiella* uropathogenesis and reveal candidate therapeutic targets.

## INTRODUCTION

Urinary tract infections (UTIs) are one of the most common bacterial infections, with over 400 million infections and over 230,000 deaths occurring annually (1, 2). The impact of UTIs is particularly significant for women, with over 50% being diagnosed with at least one UTI in their lifetime (3). With an estimated 25–40% of primary care antibiotic courses being prescribed to treat UTIs, the rise in antibiotic resistance threatens our ability to treat these common infections (4). Therefore, it is pertinent to define the bacterial mechanisms used to cause UTIs.

*Klebsiella pneumoniae* frequently causes UTIs and is the second and third leading global cause of uncomplicated and complicated UTIs, respectively (5, 6). The World Health Organization (WHO) lists *K. pneumoniae* as the top bacterial threat on the WHO Bacterial Priority Pathogens List, partially due to its accumulation of multi-drug resistance genetic determinants (7). Despite causing a variety of infections, *K. pneumoniae* is most frequently isolated in hospital settings from urine cultures (8). There are two *K. pneumoniae* pathotypes: classical (cKp) and hypervirulent (hvKp). cKp strains are nosocomial strains that primarily infect immunocompromised patients and are more prevalent in UTIs, whereas hvKp strains are community-acquired strains that can infect immunocompetent patients and often lead to more severe disease (9–11). Even though hvKp encode more virulence factors, cKp strains are more frequently isolated from UTI patients than hvKp strains (12). Despite its prevalence, cKp strains are understudied in UTIs compared to the leading cause of UTIs, uropathogenic *Escherichia coli* (UPEC) (13).

Our current understanding of uropathogenesis is primarily based on UPEC studies, which have shown that gut colonization by uropathogenic bacteria typically precedes UTIs (14). If UPEC is introduced to the periurethral area, it can colonize and ascend the urethra (14). Once UPEC traverses the urethra, it then causes ascending infections of the bladder, kidneys, and, if left uncontrolled, the bloodstream (14). Within the bladder, UPEC can invade bladder facet cells and undergo rapid intracellular replication, which results in the formation of distinctive intracellular bacterial communities (IBCs) in both humans and mouse models (15–18). Many virulence phenotypes have been characterized in UPEC, including adhesins such as type 1 and P pili, which promote UPEC adherence to epithelial cells, and immune evasion factors such as capsule, which blocks complement-mediated killing and phagocytosis (19, 20). To a lesser degree than UPEC, *K. pneumoniae* forms IBCs in murine bladders during UTIs (21). *K. pneumoniae* also encodes chaperone-usher pili systems. Although type 1 fimbriae are broadly conserved among diverse *K. pneumoniae* isolates, the *K. pneumoniae* FimH tip adhesin has a lower affinity for mannose than UPEC FimH (22, 23). In UPEC, FimH-mannose binding mediates adherence to and invasion of bladder epithelial cells via mannosylated uroplakins, driving IBC formation. The reduced mannose-binding affinity of *K. pneumoniae* FimH likely contributes to its comparatively limited IBC formation in the bladder (23–25). Nonetheless, there remains a critical knowledge gap in understanding what virulence factors cKp has compared to UPEC virulence factors and whether these phenotypes impact *in vivo* uropathogenesis.

While *K. pneumoniae* shares some virulence factors with UPEC, including capsule, *K. pneumoniae* relies more heavily on capsule for virulence and more frequently exhibits mucoidy (26). *K. pneumoniae* employs several capsule-dependent methods to evade the innate immune system, including complement resistance and reduced phagocytosis, which can be quantified by serum survival and macrophage association assays (27). Capsule serotypes K1 and K2, more often associated with hvKp strains, generally display greater virulence compared to other capsule serotypes due to enhanced immune evasion (26). In contrast to systemic infections, capsule serotypes among *K. pneumoniae* UTI strains are highly diverse, with one study reporting 23 different K serotypes from 32 UTI isolates from elderly patients (28). The mucoid phenotype is attributed to increased capsular polysaccharide (CPS) chain uniformity and length, which increase sedimentation resistance (29, 30). These long, uniform CPS chains produce tacky colonies and reduce macrophage binding (30). As such, mucoidy is typically associated with invasive hvKp infections and increased virulence; however, UTIs are predominantly caused by non-mucoid cKp strains (31). Interestingly, hvKp strains suppress mucoidy in response to urine compared to growth in Luria-Bertani (LB) broth (30). How cKp strains respond to environmental cues encountered in urine compared to hvKp remains unknown. Thus, there remains a significant gap in understanding how *K. pneumoniae* virulence factors are regulated in the context of uropathogenesis.

To examine whether cKp UTI isolates express known virulence factors, we determined if clinical cKp UTI isolates change their mucoidy and capsule abundance based on environmental cues relative to two hvKp controls. We used urine to simulate the bladder environment and LB medium for a nutrient-rich control environment. We then examined how exposure to both media impacts other urovirulence-associated phenotypes, including hemagglutination, serum resistance, and adherence to bladder epithelial cells. Ultimately, we assessed the *in vivo* fitness of four strains, which expressed varying combinations of virulence phenotypes. We found that *K. pneumoniae* clinical isolates exhibited heterogeneous fitness phenotypes that were primarily attributed to inter-strain variation and not differentially regulated between LB and urine. However, we consistently observed that urine upregulated capsule abundance, which correlated with increased serum resistance. Despite broad phenotypic heterogeneity, clinical *K. pneumoniae* strains all established robust UTI in a murine model, consistently causing pyelonephritis. This study highlights the heterogeneity of *K. pneumoniae* clinical UTI isolates and advances our understanding of *K. pneumoniae* uropathogenesis.

## RESULTS

### Genomic analyses of clinical *Klebsiella* UTI isolates

Clinical isolates were collected from patients at the University of Michigan Medical Center (32–34). Patients colonized by *Klebsiella* based on a rectal swab were followed longitudinally for subsequent clinical culture of *Klebsiella* isolates. Patients with positive urine cultures and symptoms meeting CDC National Healthcare Safety Network UTI case definitions were diagnosed with an active UTI (32, 35). Twenty-five *Klebsiella* isolates that met the clinical definitions for UTI were selected for genomic and phenotypic analyses and served as cKp strains (Table 1 and Table S1). Two model hvKp strains, KPPR1 and NTUH-K2044, were included as points of comparison to the classical strains selected for this study.

**TABLE 1:** Genomic characterization of clinical *Klebsiella* UTI isolates^*a*^

Strain	Species	Sequence type	K locus	Predicted capsule type	O locus	Predicted O type	Antibiotic resistances	Virulence score	Hypermucoidy (RmpADC/*rmp*A2)	Plasmid types	Adhesins (ECC/ECI/MS2027)
KPPR1	*K. pneumoniae*	493	KL2	K2	O1/O2v1	O1	Penicillin [SHV-1 (homolog)]	1—Yersiniabactin only	rmp 3; ICEKp1/-	None	FimA
											PapA
											SfaA
											MrkA
NTUH-K2044	*K. pneumoniae*	23	KL1	K1	O1/O2v2	O1	Penicillins (SHV-11)	4—Aerobactin and/or salmochelin with yersiniabactin (without colibactin)	rmp 1; KpVP-1 (truncated), rmp 3; ICEKp1 (truncated)/rmpA2_3–47%	IncHI1B(pNDM-MAR)/repB_KLEB	FimA
											PapA
											SfaA
											MrkA
664	*K. pneumoniae*	37	KL118	Unknown	Unknown (OL101)	Unknown (OL101)	Aminoglycosides (aadA2)	0	-/-	IncFIB(K)	FimA
							Cephalosporins (SHV-2A)			Col440l	PapA
							Phenicols [cmlA4 (homolog)]				SfaA
							Sulfonamides (sul1, sul2)				MrkA
							Trimethoprim [dfrA15 (homolog)]				
714	*K. pneumoniae*	280	KL23	K23	O1/O2v2	O2afg	Penicillins (SHV-27)	0	-/-	IncFIB(K)	FimA
										IncFII(K)	PapA
											SfaA
											MrkA
3557	*K. pneumoniae*	20	KL28	K28	O1/O2v2	O1	Penicillins (SHV-187)	0	-/-	IncFIB(K)	FimA
										IncFII(K)	PapA
											SfaA
											MrkA
4773	*K. pneumoniae*	*80c3	KL28	K28	OL104	Unknown (OL104)	Penicillins (SHV-60)	0	-/-	None	FimA
											PapA
											SfaA
											MrkA
4831	*K. pneumoniae*	1083	KL132	Unknown	O1/O2v1	O1	Penicillins (SHV-1)	0	-/-	IncFIB(K)	FimA
							Tetracycline [tet(D)]				PapA
											SfaA
											MrkA
5022	*K. pneumoniae*	628	KL123	Unknown	O3b	O3b	Penicillins (SHV-1)	0	-/-	IncFIB(K)	FimA
											PapA
											SfaA
											MrkA
5256	*K. pneumoniae*	36	KL102	Unknown	O1/O2v2	O2afg	Penicillins (SHV-11)	0	-/-	IncFIB(K)(pCAV1099-114)	FimA
										Col(pHAD28)	PapA
											SfaA
											MrkA
5712	*K. pneumoniae*	45	KL24	K24	O1/O2v1	O2a	Penicillins (SHV-1)	1—Yersiniabactin only	-/-	IncFIB(pKPHS1)	FimA
											PapA
											SfaA
											MrkA
6486	*K. variicola*	925	KL113	Unknown	O3/O3a	O3/O3a	Penicillins (LEN-2)	0	-/-	None	FimA
											PapA
											SfaA
											MrkA
6918	*K. pneumoniae*	13	KL30	K30	O1/O2v1	O1	Penicillins (SHV-1)	0	-/-	IncFIB(K)	FimA
										Col(pHAD28)/Col440II	PapA
											SfaA
											MrkA
7191	*K. pneumoniae*	2425	KL7	K7	O3b	O3b	Penicillins (SHV-11)	0	-/-	None	FimA
											PapA
											SfaA
											MrkA
7637	*K. pneumoniae*	307	KL102	Unknown	O1/O2v2	O2afg	Aminoglycosides [aac(3)-Iia, strA, strB]	0	-/-	Col(pHAD28)	FimA
							Cephalosporins [3rd Gen(CTX-M-15)]				PapA
							Fluoroquinolones (qnrB1, GyrA-83I, ParC-80I)				SfaA
							Penicillins (TEM-1D, SHV-28)				MrkA
							Sulfonamides (sul2)				
							Tetracycline [tet(A)]				
7951	*K. pneumoniae*	1380	KL23	K23	Unknown (OL102)	Unknown (OL102)	Penicillins (SHV-26)	0	-/-	IncFIB(K)	FimA
										IncFII(K)	SfaA
										Col440II	MrkA
8039	*K. pneumoniae*	253	KL112	Unknown	O1/O2v2	O1	Penicillins (SHV-36)	0	-/-	Col(pHAD28)	FimA
										IncFIB(K)	PapA
										IncFII(K)	SfaA
										Col440I	MrkA
										Col440II	
										IncFIB(pNDM-Mar)	
8341	*K. pneumoniae*	4073	KL5	K5	O1/O2v1	O1	Penicillins (SHV-37)	0	-/-	None	FimA
											PapA
											SfaA
											MrkA
8715	*K. pneumoniae*	188	KL122	Unknown	O1/O2v2	O2afg	Penicillins (SHV-37)	0	-/-	IncFII(K)	FimA
										IncFIB(K)	PapA
										Col440I	SfaA
											MrkA
10072	*K. pneumoniae*	784	KL146	Unknown	O1/O2v2	O1	Penicillins (SHV-1)	0	-/-	IncFIB(K)(pCAV1099-114)	FimA
							Fluoroquinolones (GyrA-83Y)			IncN	PapA
											SfaA
											MrkA
10079	*K. variicola*	595	KL16	K16	O5	O5	Penicillins (LEN-16)	1—Yersiniabactin only	-/-	IncFIB(K)(pCAV1099-114)	FimA
											PapA
											SfaA
											MrkA
10081	*K. variicola*	616	KL54	K54	O5	O5	Penicillins (LEN-9)	0	-/-	IncFII(K)	FimA
										IncFIA(pBK30683)	PapA
										Col440I	SfaA
											MrkA
10403	*K. quasipneumoniae*	*4562	KL101	Unknown	O3/O3a	O3/O3a	Penicillins (OKP-A-11)	0	-/-	None	FimA
											PapA
											SfaA
											MrkA
10856	*K. pneumoniae*	152	KL46	K46	O3b	O3b	Penicillins (SHV-1)	0	-/-	IncFIB(K)	FimA
											PapA
										IncFIA(pBK30683)	SfaA
											MrkA
11465	*K. pneumoniae*	3096	KL58	K58	O3b	O3b	Aminoglycosides [aac(6′)-Ib-cr, strA, strB]	0	-/-	IncI1-I(Alpha)	FimA
							Cephalosporins [3rd Gen(CTX-M-15)]			IncFIA(HI1)	PapA
							Fluoroquinolones (qnrB1)			IncFIB(K)	SfaA
							Penicillins (OXA-1, TEM-1D, SHV-26)			Col(pHAD28)	MrkA
							Sulfonamides (sul2)				
							Tetracycline [tet(A), tet(B)(homolog), tetR]				
							Trimethoprim [dfrA14 (homolog)]				
11469	*K. pneumoniae*	253	KL30	K30	O1/O2v2	O2afg	Penicillins (SHV-36)	0	-/-	IncFIB(K)	FimA
										IncFII(K)	PapA
										Col(pHAD28)	SfaA
										Col(pHAD28)	MrkA
11997	*K. pneumoniae*	391	KL30	K30	O1/O2v2	O1	Aminoglycosides [aac(6′)-Ib-cr, strA, strB]	1—Yersiniabactin only	-/-	IncFIB(K)/IncFII(K)	FimA
							Cephalosporins [3rd Gen(CTX-M-15)]			ColRNAI	PapA
							Fluoroquinolones (qnrB1)				SfaA
							Penicillins (OXA-1, TEM-1D, SHV-26)				MrkA
							Sulfonamides (sul2)				
							Tetracycline [tet(A)]				
							Trimethoprim [dfrA14 (homolog)]				
12022	*K. pneumoniae*	5142	KL27	K27	O3b	O3b	Penicillins [SHV-187 (homolog)]	0	-/-	None	FimA
											PapA
											SfaA
											MrkA

^*a*^ Genomes were assembled in PATRIC, then uploaded to pathogenwatch for species identification and characterization. Isolates with ≥25% identity to major pilin subunit sequences present in UPEC are listed. A dash (-) indicates the absence of the noted gene.

Genomic analyses of the UTI isolates using Pathogenwatch revealed that 21 UTI isolates were *K. pneumoniae* subsp. *pneumoniae*, three isolates (Kp6486, Kp10079, and Kp10081) were *K. pneumoniae* subsp. *variicola*, and one isolate (Kp10403) was *K. pneumoniae* subsp. *quasipneumoniae* (Table 1) (36–38). According to Pathogenwatch, all *K. pneumoniae* UTI isolates had low virulence scores (0–1) and did not encode the *rmp* locus (Table 1). The *rmp* locus is often encoded by hvKp strains and is responsible for increased capsule production and mucoidy (39). Although *K. pneumoniae* often encode multiple chaperon-usher pili loci, we focused on examining the conservation of the most well-characterized pili (24). The conservation of common pili was identified in the *Klebsiella* UTI isolates by performing BLAST analyses using major subunit sequences from UPEC strains (40). All isolates, except Kp7951, encoded FimA homologs with 81–83% identity, PapA homologs with 25–31% identity, SfaA homologs with 57–65% identity, and MrkA homologs with 93–95% identity to UPEC major subunit sequences (Tables 1 and 2). Kp7951 encodes an FimA homolog with 30–32% identity, no PapA homolog, an SfaA homolog with 35% identity, and a MrkA homolog with 26% identity to UPEC. Overall, BLAST analysis revealed that cKp isolates had high major subunit homology with UPEC.

### Culturing *K. pneumoniae* in urine broadly increases capsule abundance

Recent studies observed that nutrient-limited conditions promote cKp and hvKp capsule retention compared to rich culture conditions and that hvKp strains increased capsule abundance when cultured in human urine (30, 41). Therefore, we sought to determine if cKp strains also increased capsule abundance in nutrient-limited conditions. We cultured the 25 cKp UTI isolates in sterile-filtered pooled human urine or LB medium (Table 1) (32–34). Cell-associated capsule abundance was quantified by measuring uronic acid content in crude capsule extracts (42).

**Fig 1:**
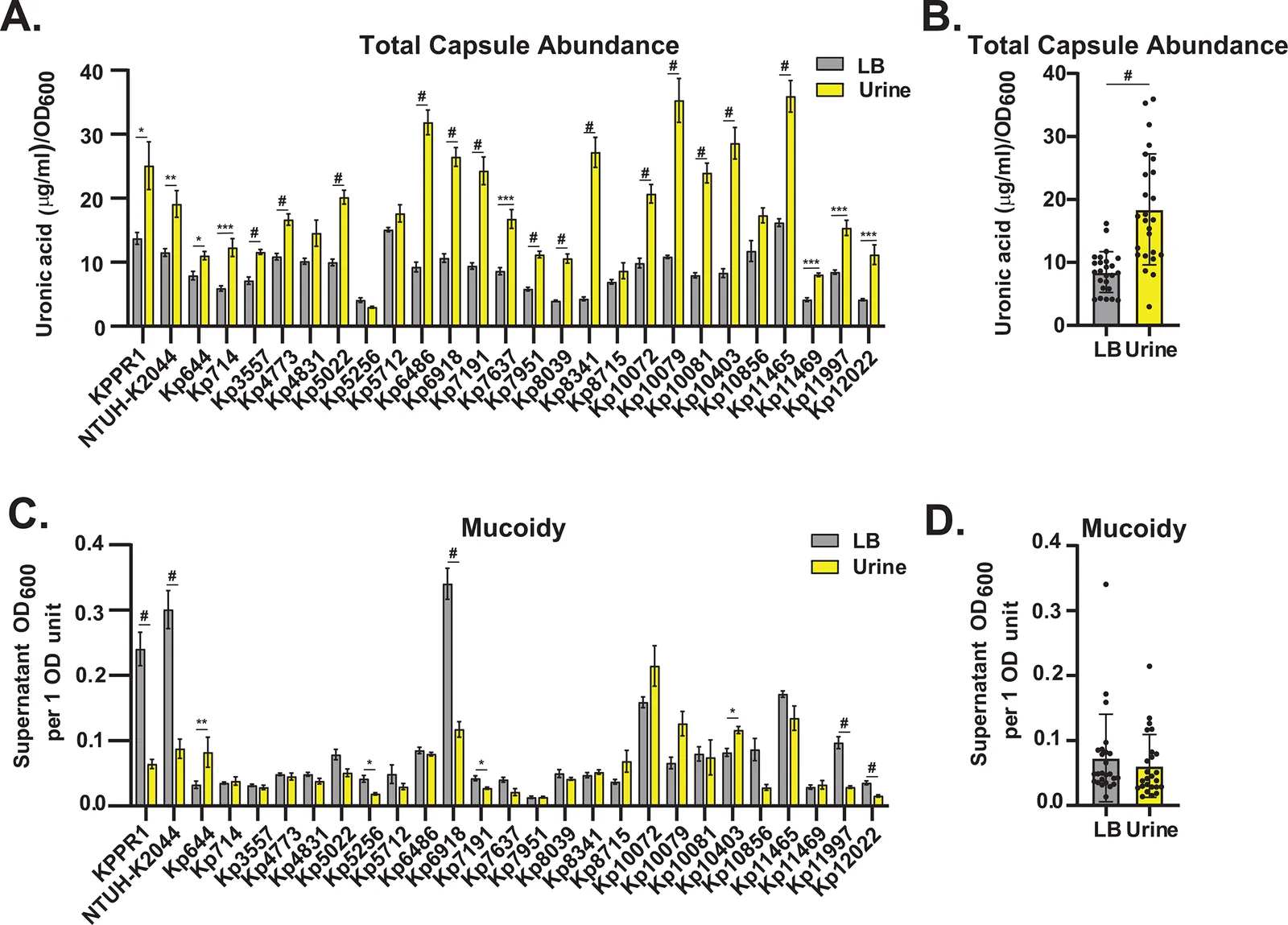
Culturing *K. pneumoniae* in urine increases capsule abundance but exerts heterogeneous effects on mucoidy. Two hvKp strains (KPPR1 and NTUH-K2044) and 25 cKp UTI isolates were cultured in LB medium or in sterile-filtered pooled female human urine. (**A**) Capsule abundance was quantified by measuring uronic acid content in crude capsule extracts. In brief, bacterial cell pellets were washed with PBS, capsule was purified from the cell surface, and then uronic acid content was quantified and normalized to the culture OD_600_. (**B**) Mean capsule abundance of each clinical UTI isolate in panel A was plotted and compared between LB medium and urine. (**C**) Mucoidy was determined by quantifying the supernatant OD_600_ after sedimenting 1 OD_600_ unit of culture at 1,000 *× g* for 5 min. (**D**) The mean sedimentation resistance of each clinical UTI isolate in panel C was plotted and compared between LB medium and urine. (**A and C**) Data presented are the mean of experiments performed ≥3 independent times, each with biological triplicates. (**B and D**) Each dot represents the mean of one strain in panel A or C. Error bars represent the standard error of the mean. (**A and C**) Statistical significance was determined using an unpaired *t*-test with Holm-Šídák post-test or (**B and D**) a paired Student’s *t*-test (*P < 0.05; **P < 0.01; ***P < 0.001; and ^#^*P* < 0.0001).

As predicted, cKp UTI isolates cultured in urine generally had higher capsule abundance relative to LB medium (Fig. 1A and B). However, five UTI isolates (Kp4831, Kp5256, Kp5712, Kp8715, and Kp10856) did not have increased capsule abundance in urine (Fig. 1A). Regardless of culture condition, hvKp isolates, KPPR1 and NTUH-K2044, produced similar amounts of capsule compared to cKp isolates, reinforcing recent reports that hvKp isolates do not necessarily produce more capsule than cKp isolates (Fig. S1) (43, 44). In addition, despite urine generally increasing capsule production, all hvKp and cKp strains grew significantly better in LB compared to urine (Fig. S2). Thus, increased capsule abundance in urine was not driven by increased growth relative to LB. Altogether, these data support that capsule abundance is elevated in nutrient-limited environmental conditions like urine, although some strain-dependent variations occur.

### Mucoidy varies between strains and culture conditions

Recent data have established that CPS chain length drives mucoidy, not total capsule abundance (29, 30, 39, 45). Furthermore, we have reported that hvKp mucoidy is regulated by environmental signals; for example, human urine suppresses mucoidy in hvKp strains (30). Interestingly, there have been sporadic reports of cKp strains appearing mucoid in clinical isolates, including from bloodstream infections (43, 46, 47). Thus, we hypothesized that some cKp UTI isolates may also be mucoid, and that mucoidy in cKp may also be regulated in response to urine environmental cues.

We used a standard sedimentation assay to quantify mucoidy, where increased sedimentation resistance indicated increased mucoidy (42). Historically, hvKp strains have greater mucoidy than cKp strains, which lack the *rmp* operon (48). We found that the hvKp strains, KPPR1 and NTUH-K2044, behaved as expected, with greater mucoidy than most cKp strains in LB medium, which decreased when the bacteria were cultured in urine (Fig. S1B) (30). Surprisingly, some cKp UTI isolates exhibited mucoidy; however, the cKp UTI isolates did not consistently increase or decrease mucoidy when cultured in human urine relative to LB broth (Fig. 1C and D).

Notably, none of the cKp strains encode the hypervirulence-associated *rmp* locus, which is known to regulate mucoidy in hvKp strains (Table 1; Fig. S3) (30, 31, 39). However, spontaneous mutations in the Wzc capsule chain length regulator have also been reported to induce mucoidy in both cKp and hvKp isolates (Fig. S3, legend) (30, 31). To examine whether any known mucoidy-inducing Wzc variants were present in the mucoid UTI isolates, we aligned the Wzc protein sequences from strains Kp6918, Kp10072, Kp10079, Kp10403, Kp10856, and Kp11465 to KPPR1 Wzc using Clustal Omega (Fig. S3) (30). While no Wzc variants known to confer mucoidy were identified, Kp11465 had a Ser139Ile variant. Previously, variants at Leu73Phe and Ser139Leu were co-isolated in a mucoid cKp strain; it is unclear whether Wzc Ser139Ile is sufficient to cause Kp11465 mucoidy by only converting the serine residue to isoleucine (30, 31, 39, 49). Since all except one mucoid cKp strains did not appear to have mucoidy-inducing Wzc variants, we propose that some cKp strains may encode other gene(s) that confer mucoidy, and these genes are likely regulated differently than the *rmp* locus in hvKp strains (47). Finally, comparing mean cKp strain mucoidy to hvKp strains confirmed that cKp strains are generally less mucoid than hvKp strains when cultured in LB medium (Fig. S1B) (43).

### Despite encoding pili, *K. pneumoniae* UTI isolates rarely hemagglutinate red blood cells

We examined the distribution and homology of common UTI-associated pili in our panel of 25 cKp UTI isolates. All isolates encoded homologs to common UTI-associated major pilin subunits: FimA, SfaA, and MrkA (Table 1). Furthermore, all isolates encoded a PapA homolog, except Kp7951. Adhesin binding can be detected using hemagglutination assays. Over 70% of UPEC isolates hemagglutinate guinea pig red blood cells (RBCs) via mannose-dependent adhesins like FimH (50). Thus, we examined the ability of each strain to hemagglutinate guinea pig RBCs and assessed if pili were regulated in response to human urine.

**Fig 2:**
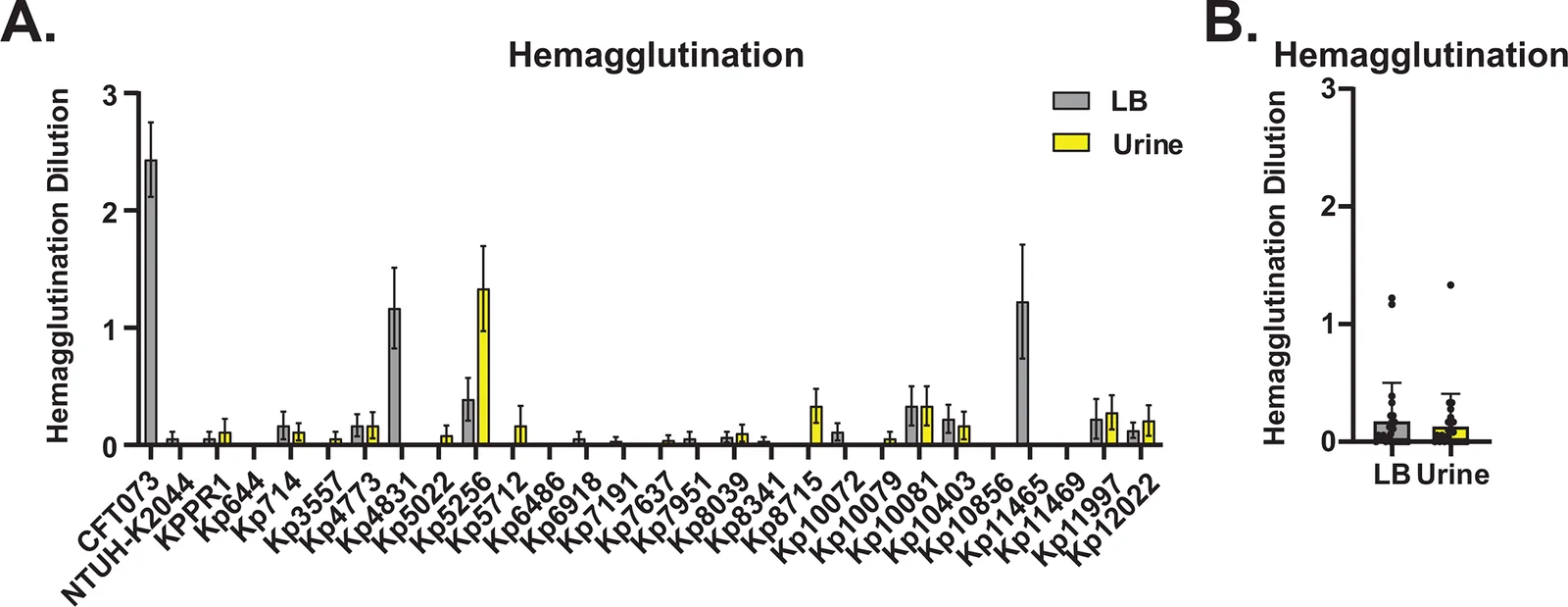
*K. pneumoniae* UTI isolates weakly and rarely hemagglutinate guinea pig red blood cells (RBCs). Twenty-five *K. pneumoniae* clinical UTI isolates and two hvKp laboratory strains, KPPR1 and NTUH-K2044, were cultured in either LB medium or sterile-filtered pooled human urine, then serially diluted 1:1 with guinea pig RBCs and incubated to assess hemagglutination. (**A**) The dilution at which hemagglutination occurred was blindly scored and averaged. UPEC strain CFT073 was used as a positive control. Data presented are the mean of experiments performed ≥3 independent times, in triplicate. (**B**) Mean hemagglutination of each clinical strain was plotted and compared between LB medium and urine. Statistical significance was assessed using (**A**) an unpaired *t*-test with Holm-Šídák post-test or (**B**) a paired Student’s *t*-test. (**A and B**) Error bars represent standard error of the mean.

We statically cultured the 25 cKp UTI isolates, two hvKp lab strains, and one UPEC strain, CFT073, in LB medium or urine for 72 h. Bacteria were suspended in PBS, then incubated with guinea pig RBCs for 1 h, and then assay images were blindly scored (51). Three cKp isolates weakly hemagglutinated guinea pig RBCs in a culture-dependent manner (Fig. 2A). Although not statistically significant, Kp4831 and Kp11465 only hemagglutinated guinea pig RBCs when cultured in LB medium, while Kp5256 hemagglutinated red blood cells more when pre-cultured in urine (Fig. 2A). When comparing all cKp UTI isolates, there was no significant difference in hemagglutination after growth in LB medium vs urine (Fig. 2B). Furthermore, no differences in hemagglutination were observed between hvKp or cKp strains in either medium (Fig. S1C). Notably, even the cKp UTI isolates that did hemagglutinate guinea pig RBCs were less effective than UPEC strain CFT073 (Fig. 2A). Altogether, these data reveal that *K. pneumoniae* adhesins do not hemagglutinate guinea pig RBCs well, suggesting that cKp adhesins may not be expressed under the tested conditions or may not strongly bind moieties on the guinea pig RBC surface.

### Culturing UTI isolates in urine increases complement resistance

**Fig 3:**
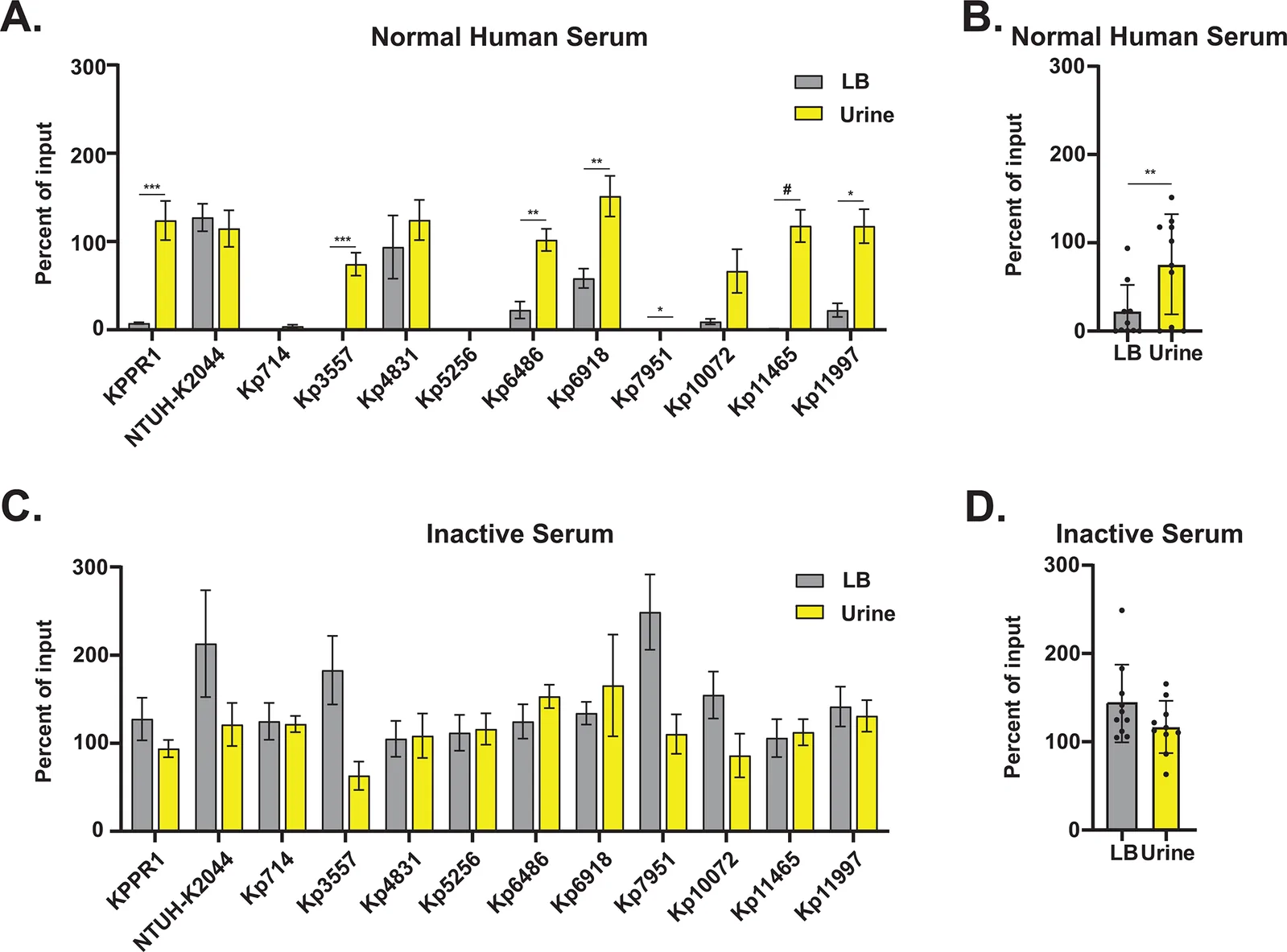
*K. pneumoniae* are more resistant to complement-mediated killing when cultured in urine. Ten *K. pneumoniae* cKp UTI isolates, as well as two hvKp laboratory strains, KPPR1 and NTUH-K2044, were cultured in either LB medium or sterile-filtered pooled human urine, washed in PBS, and then 1 × 10^5^ CFU/mL were co-incubated with (**A and B**) 90% active normal human serum or (**C and D**) 90% heat-inactivated normal human serum at 37°C for 90 min. The percent survival was calculated by dividing the output CFUs by input CFUs for each strain. Mean serum resistance of each clinical UTI isolate in (**B**) 90% active normal human serum or (**D**) 90% heat-inactivated normal human serum was plotted and compared between LB medium and urine. (**A and C**) Data presented are the mean of experiments performed ≥3 independent times, each in triplicate. (**B and D**) Each dot represents the mean of one strain in panel A or C. Statistical significance was determined using (**A and C**) an unpaired *t*-test with Holm-Šídák post-test or (**B and D**) a paired Student’s *t*-test (*P < 0.05; **P < 0.01; ***P < 0.001; and ^#^*P* < 0.0001). (**A–D**) Error bars represent standard error of the mean.

Human serum resistance measures the ability of bacteria to evade complement-mediated bactericidal activity, an important virulence factor for bacteria during urosepsis (52, 53). Both hvKp strains and a sub-set of the cKp UTI isolates were cultured in LB broth or urine prior to human serum exposure to compare the effect of growth environment on complement susceptibility. Urine collected from healthy individuals contains undetectable levels of complement activation products, minimizing the likelihood that urinary complement confounds the serum sensitivity assay (54). The subset of clinical isolates (*N* = 10) represented a variety of mucoidy, capsule abundance, and hemagglutination phenotypes quantified in Fig. 1 and 2. Serum resistance was determined by incubating 2 × 10^6^ CFU of bacteria in 90% active serum for 90 min (Fig. 3A and B). Output CFUs were enumerated and normalized to input CFUs. Heat-inactivated serum was tested in parallel as a negative control (Fig. 3C and D). A percent survival less than 100% indicates susceptibility to complement-mediated killing.

Most cKp UTI isolates, as well as KPPR1, exhibited increased complement resistance when cultured in urine compared to LB medium (Fig. 3B). NTUH-K2044, Kp714, Kp4831, Kp5256, and Kp10072 did not have a significant difference in serum resistance between the two conditions, despite NTUH-K2044, Kp714, and Kp10072 having increased capsule in urine (Fig. 1A and 3A). NTUH-K2044 and Kp4831 had high survival in both LB and urine, suggesting that some strains are intrinsically more resistant to complement, independent of capsule abundance (Fig. 3A). Notably, Kp5256 (likely acapsular), Kp7951, and Kp714 had practically no serum survival in either condition (Fig. 3A). Overall, the cKp strains displayed a significant increase in bacterial survival when cultured in urine (Fig. 3B and Fig. S1D). As expected, all isolates survived in heat-inactivated serum regardless of culture condition (Fig. 3C and D). Combined, these data indicate that relative to LB medium, culturing cKp UTI isolates in urine increases resistance to complement-mediated killing, likely due to increased capsule abundance.

### Bladder cell association and invasion are driven by strain differences, not environmental conditions

**Fig 4:**
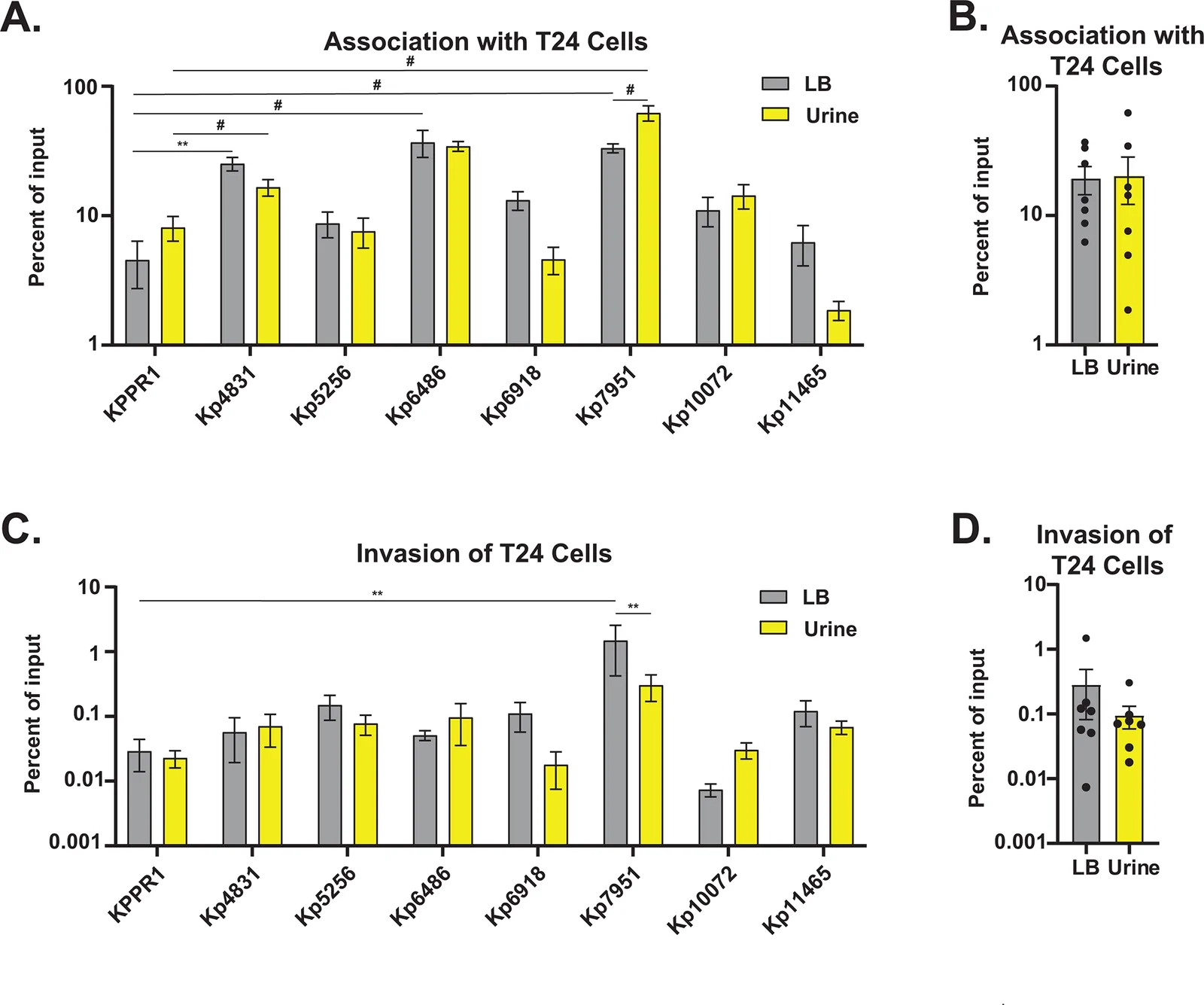
Bladder cell association and invasion are strain-dependent and not influenced by urine. Seven cKp UTI isolates and hvKp strain, KPPR1, were cultured overnight in either LB medium or sterile-filtered pooled human urine. The bacterial cells were washed with PBS and then resuspended in McCoy medium. T24 bladder cells were infected with each strain at an MOI of 10 for 2 h, followed by an incubation at 4°C for 1 h (**A and B**) without treatment or (**C and D**) with 100 μg/mL gentamicin. Samples were washed three times with PBS and then lysed with Triton X-100. Input and cell-associated bacterial colony-forming units (CFUs) were enumerated on LB agar. (**A and C**) Data presented are the log of the mean of experiments performed ≥ 3 independent times, each in triplicate. (**B and D**) Each dot represents the mean of one strain in A or C. Error bars represent the standard error of the mean. Statistical significance was determined using (**A and C**) two-way ANOVA with Bonferroni post-test or (**B and D**) a paired Student’s *t*-test (**P < 0.01 and #*P* < 0.0001).

We then modeled cKp bladder cell association and invasion using T24 bladder cells to determine whether strain differences or environmental conditions might impact bladder colonization. From the isolates used in Fig. 3, we further down sampled to seven clinical UTI isolates and one hvKp strain, still encompassing a variety of quantified phenotypes (Fig. 1 to 3). Despite invasion occurring at a low frequency, there were some association and invasion differences observed between strains (Fig. 4C). Only Kp7951 exhibited a significant difference in T24 bladder cell association or invasion after culturing in urine relative to LB medium (Fig. 4A and C). Compared to KPPR1, Kp7951 and Kp6486 both bound bladder cells more after growth in either culture condition, while Kp4831 only bound bladder cells more after culture in LB medium (Fig. 4A). Kp7951 also exhibited a significant increase in bladder cell invasion compared to KPPR1 after culture in LB medium (Fig. 4C). Although there was a trend toward cKp exhibiting increased bladder cell association and invasion compared to hvKp, there was no significant difference (Fig. S1E through F). Additionally, we calculated the ratio of intracellular bacteria relative to extracellular-associated bacteria to quantify invasion efficiency for each strain (Fig. S4). While not statistically significant, Kp5256 and Kp11465 had the highest internalization efficiency, while Kp4831 had the lowest internalization efficiency. Finally, there was no overall medium-dependent difference in bladder cell association or invasion (Fig. 4B and D).

### Capsule abundance and serum resistance are correlated

**Fig 5:**
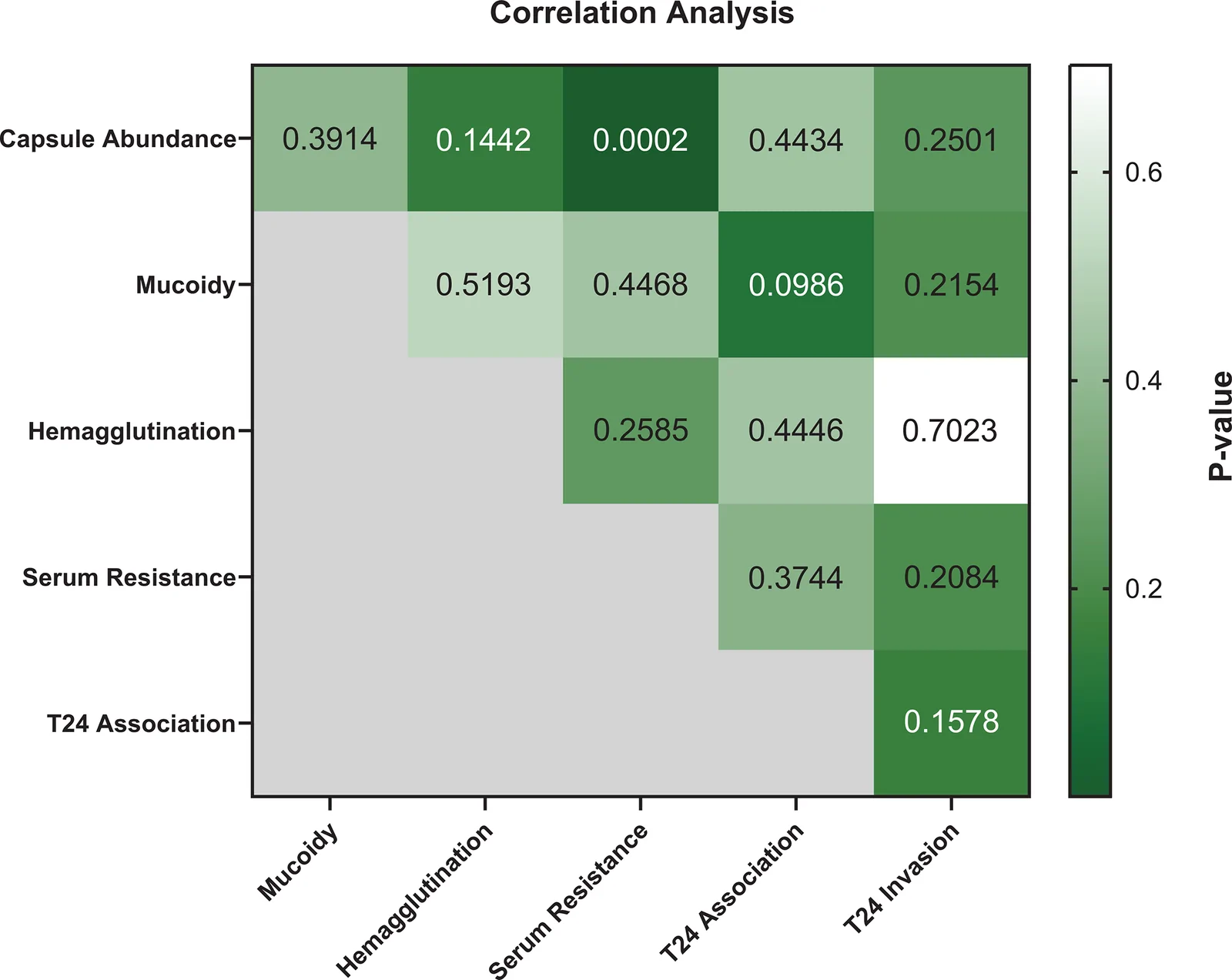
Correlation analyses between quantified *K. pneumoniae* fitness phenotypes. A correlation matrix was used to compare relations between the virulence phenotypes quantified in Fig. 1 to 4 using a nonparametric Spearman correlation and two-tailed *P* values with a 95% CI. The *P* values are displayed, and *P* < 0.05 was deemed significant. The lighter shade indicates less significant correlation, while the darker green indicates more significant correlation.

Using a correlation analysis matrix, we tested if there were relationships between the various virulence phenotypes by combining data collected for all strains in both culture conditions (Fig. 5). We only observed a significant correlation between capsule abundance and serum resistance (*P* = 0.0002). These data align with prior observations that increased capsule presence promotes complement resistance (55, 56). While not significant, mucoidy tended to correlate with T24 association (*P* = 0.09858), which aligns with prior reports that mucoidy interferes with bacterial binding to host cells (43, 45). Interestingly, hemagglutination overall had a very low correlation with other virulence factors. Thus, most quantified phenotypes did not appear to be related to each other, except that elevated capsule production resulted in greater protection from complement-mediated killing.

### All cKp UTI isolates establish pyelonephritis, despite phenotypic heterogeneity

**Fig 6:**
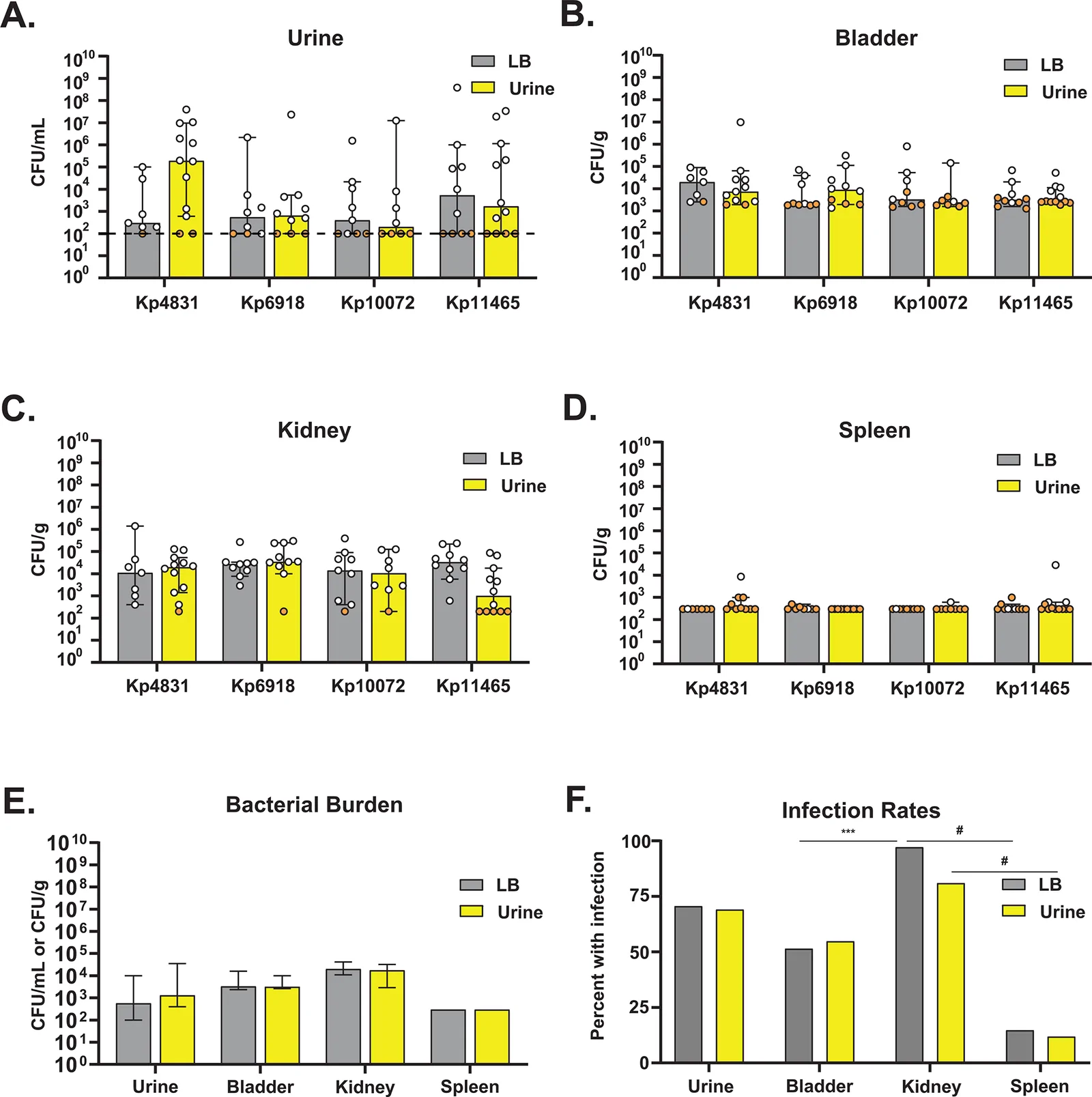
Despite phenotypic heterogeneity, cKp UTI isolates exhibit kidney tropism. Four UTI isolates were cultured in either LB medium or sterile-filtered pooled human urine, then washed and resuspended in PBS prior to transurethral inoculation into female CBA/J mice. After 48 h, the bacterial burdens in the (**A**) urine, (**B**) bladder, (**C**) kidneys, and (**D**) spleens were enumerated. Each dot represents an individual mouse, the dashed line represents the limit of detection (100 CFU/mL), and the orange dots indicate mice with no recoverable bacteria in the outputs. (**E**) The combined median bacterial burden of the four strains from panels A to D was calculated for each organ or urine. (**F**) Organs and urine with no recoverable CFUs in panels A–D were classified as “uninfected,” and the percent infected was calculated. Infections with each strain were performed on at least two independent days. Statistical significance was determined using two-way ANOVA with Tukey’s post-test (***P < 0.001; ^#^*P* < 0.0001). (**A–E**) Error bars represent standard error of the mean.

Four strains representing a variety of phenotypes were selected to assess *in vivo* fitness in a CBA/J murine UTI model. For example, all four strains significantly increased capsule abundance in urine compared to LB medium, but Kp4831 and Kp11465 hemagglutinated guinea pig RBCs while Kp6918 and Kp10072 did not (Fig. 1B). *K. pneumoniae* strains were cultured in either LB or urine, then washed in PBS, and 10^8^ CFUs were transurethrally instilled into female CBA/J mice. CBA/J mice were chosen due to their use as an established model of ascending UTIs (57–60). After 48 h, bacterial burdens in the bladder, kidneys, spleen, and urine were enumerated. There was no significant difference in bacterial burdens between strains at each site, nor were there significant differences between culture conditions prior to infection (Fig. 6A through D). When the data from the four strains were combined, there were no significant differences in median bacterial burdens between host sites (organ or urine) or culture conditions (Fig. 6E). However, when the frequency of recoverable CFUs from each host site was quantified, there were significant differences in the percentage of infected organs across the host sites (Fig. 6F). Specifically, all *K. pneumoniae* strains colonized kidneys more frequently than either bladders or spleens. Spleens were rarely colonized with bacteria (<15%), suggesting that *K. pneumoniae* infected the kidneys via the ascending route rather than hematogenous spread. These data reveal that despite being phenotypically diverse, the four cKp clinical UTI isolates all successfully colonized the urinary tract, predominantly establishing pyelonephritis.

## DISCUSSION

Despite being the second leading cause of UTIs, *K. pneumoniae* uropathogenesis is understudied relative to UPEC. Although *K. pneumoniae* employs some of the same virulence factors as UPEC, it relies more heavily on capsule for virulence and more frequently exhibits mucoidy. We found that while some fitness-associated phenotypes were differentially expressed based on environmental cues, many phenotypes were expressed in a strain-dependent manner, i.e., there were no consistent differences between growth media; rather, phenotypes differed between strains. Despite the observed heterogeneity amongst *K. pneumoniae* clinical UTI isolates, strains were able to establish UTIs *in vivo*, regardless of virulence phenotype expression. Interestingly, *K. pneumoniae* strains all had robust kidney colonization, suggesting an unknown conserved bacterial property that promotes UTI ascension.

To examine the genomic properties encoded by *K. pneumoniae* clinical UTI isolates, we analyzed 25 clinical UTI isolates using Pathogenwatch and performed BLAST analyses with known major subunit sequences (40). The *K. pneumoniae* clinical UTI isolates had high major subunit homology to UPEC, with all but one strain encoding FimA homologs with 81–83% identity, PapA homologs with 25–31% identity, SfaA homologs with 57–65% identity, and MrkA homologs with 93–95% identity (Table 1). The other strain, Kp7951, encoded all homologs to a lesser degree but did not encode a PapA homolog. Thus, *K. pneumoniae* encodes many of the same pili as UPEC, including type 1 fimbriae, which primarily mediate bladder cell adherence and invasion (61, 62). In agreement with our bioinformatic analysis, we found that *K. pneumoniae* clinical isolates adhered to and invaded T24 bladder cells, with strain-dependent differences (Fig. 4) (61). One limitation of the T24 cell line, like most bladder cell lines, is its inability to differentiate into uroplakin-positive urothelial cells (63). Uroplakins are transmembrane proteins present on urothelial superficial umbrella cells that serve as receptors for type 1 pili in UPEC (64–66). Yet even in the absence of uroplakins, *K. pneumoniae* adheres to and invades T24 cells. This suggests that *K. pneumoniae* could bind to the uroplakin-negative basal and intermediate cells of the bladder underlying the umbrella cell layer (63, 66, 67).

CPSs are produced by both UPEC and *K. pneumoniae* and block innate immune responses, such as phagocytosis and complement-mediated killing (Fig. 1A) (20, 68). Serum resistance, which allows UPEC to evade complement-mediated killing, is a key virulence determinant for urosepsis (53). *K. pneumoniae*, like UPEC, also demonstrates serum resistance in a strain-dependent manner, with only three strains exhibiting little to no complement resistance (Fig. 3A). While both *E. coli* and *K. pneumoniae* produce CPS, *K. pneumoniae* controls CPS length and uniformity to generate a mucoid phenotype, which is an important virulence factor associated with invasive hvKp infections (29, 30, 39, 45). Hypermucoid phenotypes have previously been associated with hvKp isolates and more severe infections, but cKp strains can exhibit this phenotype as well (30, 43, 47). *K. pneumoniae* clinical UTI isolates produced varying levels of mucoidy, which did not correlate with culture conditions (Fig. 1C).

Hemagglutination is mediated by both type 1 and P fimbriae, with type 1 and P fimbriae being attributed to primarily supporting cystitis and pyelonephritis, respectively (69–73). UPEC-expressing adhesins are often detected using hemagglutination assays (50). Type 1 fimbriae undergo mannose-sensitive hemagglutination, while P fimbriae can be detected by mannose-resistant hemagglutination (74, 75). Hemagglutination of guinea pig erythrocytes is mediated by type 1 fimbriae and is mannose-sensitive, while hemagglutination of human erythrocytes requires both mannose-sensitive and -resistant mechanisms (50, 74–76). More virulent strains of UPEC are more likely to be inhibited by mannose; therefore, we tested *K. pneumoniae* clinical UTI isolates using a mannose-sensitive hemagglutination assay with guinea pig RBCs to determine whether, like UPEC, hemagglutination can be used to predict virulence (75). Despite all isolates encoding an FimA homolog, most *K. pneumoniae* clinical UTI isolates demonstrated poor guinea pig RBC hemagglutination (Fig. 2A). Similar to UPEC, *K. pneumoniae* encodes FimH, an important adhesin at the tip of type 1 pili (23). However, *K. pneumoniae* FimH has lower affinity for mannose than UPEC FimH and therefore hemagglutinates guinea pig RBCs poorly (22, 23). These interspecies differences in FimH-mannose affinity align with our observation that the UPEC strain CFT073 hemagglutinated guinea pig RBCs significantly better than all the *K. pneumoniae* strains (Fig. 2A).

In summary, while *K. pneumoniae* and UPEC isolates share several virulence mechanisms, including pili, bladder cell invasion and adhesion, CPS production, and serum resistance, they differ in other key aspects. *K. pneumoniae* isolates exhibit mucoidy more frequently than UPEC (though still in a minority of *K. pneumoniae* UTI isolates) and lack robust guinea pig RBC hemagglutination, whereas UPEC demonstrates strong hemagglutination but is rarely mucoid. These distinct virulence profiles suggest that *K. pneumoniae* and UPEC employ different pathogenic strategies during UTI.

In addition to observing phenotypic heterogeneity between the clinical UTI isolates, we also considered how culture media alter *K. pneumoniae* virulence phenotypes and whether those changes affect uropathogenesis. Despite previous research demonstrating that hvKp strains exhibit decreased mucoidy in human urine compared to LB medium, mucoid cKp isolates did not show a significant difference between the two culture conditions (Fig. 1C and D) (30). Moreover, the different culture conditions did not affect RBC hemagglutination or T24 cell invasion or association (Fig. 2 and 4). One limitation is that some clinical isolates alter their mucoidy in response to the bladder cell culture medium, McCoy’s medium, which may confound the bladder association data. However, as previously reported, capsule abundance tended to be elevated in strains cultured in urine compared to LB medium (Fig. 1B) (30). The increased capsule production is likely driven by the nutrient-limited conditions in urine, which has been reported elsewhere (30, 41). Capsule also protects bacteria from complement-mediated killing (55, 56). Thus, as expected, serum resistance increased when strains were cultured in urine compared to LB medium (Fig. 3B). We then employed a correlation matrix analysis to test the relationship between virulence phenotypes (Fig. 5). As expected, capsule abundance and serum resistance significantly correlated. Other work has shown that increased *K. pneumoniae* capsule production blocks complement-mediated killing and phagocytosis (20, 27, 55, 56, 68). Therefore, the urine-induced increase in capsule observed here likely not only increases serum resistance but may also protect against phagocytosis in the urinary tract, although we did not test this (68). It is well-established that neutrophil-mediated phagocytosis is critical for restricting and clearing UTIs (77, 78). Based on clinical observation, serum-resistant UTI isolates are correlated with increased incidences of pyelonephritis and bacteremia (79). Thus, our data, combined with prior reports, suggest that increased capsule production may not only increase serum resistance but also could predict increased phagocytosis resistance and propensity for ascending UTI (55, 56).

Despite displaying heterogeneous phenotypes among clinical UTI isolates, *K. pneumoniae* consistently colonized the upper urinary tract in our murine UTI model. CBA/J mice are more susceptible to ascending UTIs than other commonly used strains, such as C57BL/6, BALB/c, and C3H/HeN mice, making them a reliable model for ascending UTI (59, 80–83). Previous studies report that using a syringe pump to deliver the inocula at a constant rate, which is our standard practice, eliminates or significantly reduces vesicoureteral reflux by confirming that bacteria do not reach the kidneys passively during inoculation (59, 84). Although the CBA/J ascending UTI model was developed and validated using UPEC, the underlying susceptibility phenotype is likely applicable to other gram-negative bacteria, including *K. pneumoniae*. Together, the absence of vesicoureteral reflux under controlled inoculation and the robust susceptibility of CBA/J mice to ascending infection establish this as a tractable model for studying ascending *K. pneumoniae* UTI.

Across all infection sites, there was no significant difference in bacterial burdens between strains, organs, or pre-infection culture conditions (Fig. 6A through E). However, in both media conditions, the clinical UTI isolates colonized kidneys more frequently than bladders or spleens (Fig. 6F). We interpret the lack of spleen colonization to indicate that the bacteria reach the kidneys by ascending the ureters from the colonized bladder, rather than through hematogenous dissemination. Our *in vivo* murine data are consistent with clinical observations that *K. pneumoniae* is more likely to cause severe acute pyelonephritis compared to UPEC (85). Additionally, UTI patients infected with *K. pneumoniae* have significantly higher 30-day mortality rates compared to patients infected with UPEC (85). Our data, combined with clinical observation, suggest that *K. pneumoniae* consistently colonizes kidney tissue (85). Since this was observed in genetically and phenotypically distinct clinical isolates, this suggests that core genomic properties may underlie kidney colonization. To determine whether *K. pneumoniae* exhibit true kidney tropism, future studies would need to track the kinetics of ascension, the efficiency of kidney establishment relative to bladder colonization, and the expansion of the founding population within the kidney over time. A limitation of this model is that mice do not express globoside (GbO4), which is a glycolipid kidney epithelial receptor for UPEC Pap pili in humans (73, 86). However, previous studies report that Pap pili are not essential for UTI ascension in CBA/J mice, as other pili may substitute for Pap (73, 84). Despite these limitations, controlled transurethral instillation of *K. pneumoniae* into CBA/J mice is the most tractable current model for studying ascending UTI and provides a physiologically relevant framework for assessing kidney colonization (73, 84). Further investigation is merited to identify the bacterial and host determinants driving *K. pneumoniae-*kidney interactions and their impact on UTI severity. Understanding these host-pathogen interactions may reveal new intervention points to improve outcomes for patients experiencing a *K. pneumoniae-*incited UTI.

Characterizing this collection of cKp UTI isolates has revealed that *K. pneumoniae* uropathogenesis is likely distinct from UPEC. The data presented here demonstrate that clinical *K. pneumoniae* UTI isolates exhibit heterogeneous expression of virulence phenotypes, some of which are regulated by environmental cues. Yet, despite this heterogeneity, the strains consistently colonize the urinary tract, particularly the kidneys. These observations lead us to propose that core genome elements support *K. pneumoniae* urinary tract ascension, establishing pyelonephritis. We propose that the core genomic elements that support ascension are constitutively expressed or activated in the urinary tract. Future research defining the bacterial and host elements required for ascension to the kidneys will advance our understanding of *K. pneumoniae* uropathogenesis and may present the opportunity for improved UTI management.

## MATERIALS AND METHODS

### Strains and culture conditions

All strains described in these studies are detailed in Table 1. Bacteria were cultured in either pooled healthy female human urine or lysogeny broth (LB, 5 g/L yeast extract, 10 g/L tryptone, and 0.5 g/L NaCl) at 200 rpm and 37°C in an aerated culture tube at a 60° angle, unless otherwise noted (87). Solid medium was prepared by adding 20 g/L bacto-agar prior to autoclaving. Urine was pooled from at least five independent donors and then vacuum filter sterilized through a 0.2-µm PES membrane. Sterilized urine was stored in aliquots at −20°C, with working volumes stored at 4°C.

### Pathogenwatch

*K. pneumoniae* strains were characterized based on genomic sequence as published (87). In brief, raw sequence reads of each strain from the NCBI database were assembled into whole genomes using PATRIC (Table S1). The genomes for KPPR1 and NTUH-K2044 were obtained directly from the NCBI database. The assembled genomes were uploaded to Pathogenwatch for species identification and characterization (38, 88, 89). Major subunit sequences for FimA (c5393 and C5011), PapA (c3592, c5188, and C4895), and SfaA (C1109) were obtained from uropathogenic *Escherichia coli* (UPEC) strains *E. coli* O6:K2:H1 CFT073 (ECC) and *E. coli* O18:K1:H7 UTI89 (ECI) using the Kyoto Encyclopedia of Genes and Genomes (KEGG) database (40). A sequence for MrkA (protein ID: ABW83989.1) was obtained from UPEC strain MS2027 (90). Amino acid homology for each major subunit was compared to each *K. pneumoniae* strain using tBLASTn (Table S2). Those proteins with ≥25% identity were classified as pilin-positive in Table 1.

### Wzc alignments

The Wzc protein alignment was performed as described in reference 30. In brief, raw sequence reads of Kp10072, Kp10079, Kp10403, Kp10856, Kp11465, Kp664, and Kp6918 from the NCBI SRA database were assembled using PATRIC (Table S1) (91). The *wzc* ORF was identified by searching the assembled genomes for “tyrosine-kinase protein.” The predicted *wzc* ORF was confirmed by a BLAST search to confirm the ORF contained a tyrosine kinase. The ORFs were translated and aligned using Clustal Omega. The alignment was visualized using Jalview.

### Sedimentation assay

Sedimentation assays were used to quantify mucoidy of overnight samples as described with the following modifications (30). In brief, overnight samples equivalent to 1 OD_600_ unit of bacteria were transferred to individual 2 mL centrifuge tubes for samples incubated in LB broth and 10 mL centrifuge tubes for samples incubated in urine if the total amount of urine exceeded 2 mL. The samples were centrifuged at maximum speed for 15 min. All but 50 µL of supernatant fluid was decanted. The pellet was resuspended in 1 mL of PBS and centrifuged at 1,000 *× g* for 5 min. The optical density at 600 nm (OD_600_) of the supernatant was measured to determine the sedimentation resistance.

### Uronic acid quantification

Capsule abundance was quantified based on uronic acid content in CPS isolated from overnight cultures as previously described (30). In brief, overnight cultures containing 1 OD_600_ unit were transferred to individual 2 mL centrifuge tubes for strains grown in LB and 10 mL centrifuge tubes for strains grown in urine. The samples were washed to remove uronic content present in urine by centrifuging at maximum speed for 15 min. All but 50 µL of supernatant fluid was decanted. The pellet was resuspended, washed in 1 mL of PBS, and centrifuged at maximum speed for 15 min. After centrifugation, 750 µL of supernatant fluid was removed by pipette. Then, 50 µL of 1% Zwittergent 3-14 in 100 mM citric acid was mixed into the sample, and the pellet was resuspended by pipetting. Hereafter, the capsule was purified as previously described (30). In brief, samples were incubated at 50°C for 20 min and then centrifuged at high speed for 5 min. Capsular polysaccharides were precipitated with 80% ethanol on ice, centrifuged, and the pellets rehydrated in deionized water. Uronic acid content was quantified using 3-hydroxydiphenyl as described (45, 92).

### Growth curves

Growth curves were generated using the protocol described (87). In brief, bacterial strains were cultured overnight in triplicate in 3 mL of LB medium. The cultures were normalized to an OD_600_ of 0.001 in LB medium or urine, and then 100 µL was aliquoted into a microplate. The OD_600_ was recorded on a Biotek plate reader every 30 min for 16 h at 37°C with continuous, orbital shaking [282 rpm (3 mm)].

### Hemagglutination

Hemagglutination protocol was performed as described (51). Bacteria were statically cultured in either 3 mL of LB or pooled urine for 72 h at 37 °C. Then, 400 μL (LB) or 2 mL (urine) of the culture was centrifuged at 5,000 *× g* for 5 min, and the cells were gently washed in 300 μL of PBS. The cell suspensions were serially diluted 1:1 with PBS in round-bottom 96-well plates. Two hundred microliters of guinea pig erythrocytes (Innovative Research: IGPRBC10ML) was pelleted at 5,000 *× g* for 5 min and washed three times in 1 mL PBS. Erythrocytes were resuspended in 15 mL of PBS (final concentration 1% vol/vol), and 150 µL was distributed to each well. Bacteria and erythrocytes were gently mixed and incubated statically at room temperature for 1 h prior to imaging. To quantify, hemagglutination was blindly scored by three individuals. The dilution at which individuals identified hemagglutination was averaged for each round.

### Serum resistance

This assay was performed as described with the following adaptations (45). One milliliter of each overnight culture was centrifuged at 16,000 *× g* for 10 min, and the pellet was resuspended in 1 mL of PBS. The samples were normalized to an OD_600_ of 0.002 in 200 μL PBS to achieve a stock of 2 × 10^6^ CFU/mL. This working stock was then diluted 1:10 into 96-well plates with either pooled normal human complement serum (Innovative Research, CAT: ICSER50ML) or heat-inactivated (1 h at 58°C) normal human serum and incubated at 37°C for 90 min. Input and output CFU/mL were serially diluted 1:10 with PBS and spot plated on LB agar. Plates were incubated at 30°C until CFUs were visible. To quantify serum resistance, CFUs were averaged, and the percent survival relative to input was plotted.

### Bladder cell association and invasion

Cell association and invasion assays were performed using immortalized T24 bladder cells derived from the bladder of a female with colorectal adenocarcinoma (ATCC HTB-4). The cells were maintained in McCoy’s 5A medium with l-glutamine, sodium bicarbonate supplemented with 10% heat-inactivated fetal calf serum (Corning), 100 U/mL penicillin, and 100 µg/mL streptomycin in an atmosphere of 5% CO_2_. Confluent cells (~8 × 10^4^ cells/well) in 24-well tissue culture-treated dishes were washed three times with 1 mL of PBS, and then 1 mL of 8 × 10^5^ CFU/mL bacteria (MOI = 10) in additive-free McCoy’s 5A medium was added to each well. Samples were centrifuged at 54 *× g* for 5 min and then incubated at 37°C, 5% CO_2_ for 4 h, followed by an incubation at 4°C for 1 h with or without 100 μg/mL gentamicin. Samples were washed three times with PBS and then lysed with 1 mL of 0.4% Triton X-100 in PBS for 8 min on a rotary shaker. Input and cell-associated bacterial counts were determined by serial dilution and CFU enumeration on LB agar.

### Murine model of ascending urinary tract infection

These studies were performed and adapted as described (51, 80, 81, 93). In brief, female CBA/J mice (6–8 weeks old) from Jackson Laboratories were transurethrally inoculated with 50 μL of bacterial suspension (1 × 10^8^ CFU) delivered over 30 s using a sterile pediatric intravenous-access cannula (divested of its needle) connected to an infusion pump (Harvard Apparatus). The procedure was performed under anesthesia with ketamine/xylazine, and mice were recovered on a heated pad. Forty-eight hours post-inoculation, urine was collected, and then mice were euthanized by isoflurane overdose. Bladders, kidneys, and spleens were aseptically removed and homogenized according to the manufacturer’s instructions (Bullet Blender Gold, Next Advance). Urine and organ homogenates were serially diluted in PBS and enumerated on LB agar.

### Statistics

All *in vitro* experiments include data collected from three biological replicates performed in three independent experiments. All *in vivo* experiments were performed at least twice per strain. All statistical analyses were computed in Prism 10 (GraphPad Software, La Jolla, CA, USA). For experiments comparing multiple groups, significance was calculated using two-way ANOVA with a Bonferroni post-test to compare specific groups. A one-sample *t*-test was applied when comparing two groups or a single group to a hypothetical value of 1.00. A multiple correlation analysis was used to compare quantifiable virulence factors throughout several experiments. In all instances, results were considered significant if the *P* value was less than or equal to 0.05.

## SUPPLEMENTAL MATERIAL

Supplemental material iai.00508-26-s0001.pdf Fig. S1 to S4; Tables S1 to S3.

ASM does not own the copyrights to Supplemental Material that may be linked to, or accessed through, an article. The authors have granted ASM a non-exclusive, world-wide license to publish the Supplemental Material files. Please contact the corresponding author directly for reuse.

## References

[1] Klevens RM, Edwards JR, Richards CL, Horan TC, Gaynes RP, Pollock DA, Cardo DM. 2007. Estimating health care-associated infections and deaths in U.S. hospitals, 2002. Public Health Rep 122:160–166. doi:10.1177/003335490712200205 PMC1820440 17357358

[2] Yang X, Chen H, Zheng Y, Qu S, Wang H, Yi F. 2022. Disease burden and long-term trends of urinary tract infections: a worldwide report. Front Public Health 10:888205. doi:10.3389/fpubh.2022.888205 PMC9363895 35968451

[3] Caneiras C, Lito L, Melo-Cristino J, Duarte A. 2019. Community- and hospital-acquired *Klebsiella pneumoniae* urinary tract infections in Portugal: virulence and antibiotic resistance. Microorganisms 7:138. doi:10.3390/microorganisms7050138 PMC6560439 31100810

[4] Frimodt-Møller N, Bjerrum L. 2023. Treating urinary tract infections in the era of antibiotic resistance. Expert Rev Anti Infect Ther 21:1301–1308. doi:10.1080/14787210.2023.2279104 37922147

[5] Flores-Mireles AL, Walker JN, Caparon M, Hultgren SJ. 2015. Urinary tract infections: epidemiology, mechanisms of infection and treatment options. Nat Rev Microbiol 13:269–284. doi:10.1038/nrmicro3432 PMC4457377 25853778

[6] Timm MR, Russell SK, Hultgren SJ. 2025. Urinary tract infections: pathogenesis, host susceptibility and emerging therapeutics. Nat Rev Microbiol 23:72–86. doi:10.1038/s41579-024-01092-4 PMC13194463 39251839

[7] (IRC) ARDAIIaRC. 2024. WHO bacterial priority pathogens list, 2024: bacterial pathogens of public health importance to guide research, development and strategies to prevent and control antimicrobial resistance. Report No.: 978-92-4-009346-1. World Health Organization

[8] Gorrie CL, Mirčeta M, Wick RR, Judd LM, Lam MMC, Gomi R, Abbott IJ, Thomson NR, Strugnell RA, Pratt NF, Garlick JS, Watson KM, Hunter PC, Pilcher DV, McGloughlin SA, Spelman DW, Wyres KL, Jenney AWJ, Holt KE. 2022. Genomic dissection of *Klebsiella pneumoniae* infections in hospital patients reveals insights into an opportunistic pathogen. Nat Commun 13. doi:10.1038/s41467-022-30717-6 PMC9156735 35641522

[9] Podschun R, Ullmann U. 1998. *Klebsiella* spp. as nosocomial pathogens: epidemiology, taxonomy, typing methods, and pathogenicity factors. Clin Microbiol Rev 11:589–603. doi:10.1128/CMR.11.4.589 PMC88898 9767057

[10] Shon AS, Bajwa RPS, Russo TA. 2013. Hypervirulent (hypermucoviscous) *Klebsiella pneumoniae*: a new and dangerous breed. Virulence 4:107–118. doi:10.4161/viru.22718 PMC3654609 23302790

[11] Marr CM, Russo TA. 2019. Hypervirulent *Klebsiella pneumoniae*: a new public health threat. Expert Rev Anti Infect Ther 17:71–73. doi:10.1080/14787210.2019.1555470 PMC6349525 30501374

[12] Paczosa MK, Mecsas J. 2016. *Klebsiella pneumoniae*: going on the offense with a strong defense. Microbiol Mol Biol Rev 80:629–661. doi:10.1128/MMBR.00078-15 PMC4981674 27307579

[13] Foxman B, Brown P. 2003. Epidemiology of urinary tract infections: transmission and risk factors, incidence, and costs. Infect Dis Clin North Am 17:227–241. doi:10.1016/s0891-5520(03)00005-9 12848468

[14] Kaper JB, Nataro JP, Mobley HL. 2004. Pathogenic *Escherichia coli*. Nat Rev Microbiol 2:123–140. doi:10.1038/nrmicro818 15040260

[15] Garofalo CK, Hooton TM, Martin SM, Stamm WE, Palermo JJ, Gordon JI, Hultgren SJ. 2007. *Escherichia coli* from urine of female patients with urinary tract infections is competent for intracellular bacterial community formation. Infect Immun 75:52–60. doi:10.1128/IAI.01123-06 PMC1828379 17074856

[16] Justice SS, Hung C, Theriot JA, Fletcher DA, Anderson GG, Footer MJ, Hultgren SJ. 2004. Differentiation and developmental pathways of uropathogenic *Escherichia coli* in urinary tract pathogenesis. Proc Natl Acad Sci USA 101:1333–1338. doi:10.1073/pnas.0308125100 PMC337053 14739341

[17] Mulvey MA, Schilling JD, Hultgren SJ. 2001. Establishment of a persistent *Escherichia coli* reservoir during the acute phase of a bladder infection. Infect Immun 69:4572–4579. doi:10.1128/IAI.69.7.4572-4579.2001 PMC98534 11402001

[18] Rosen DA, Hooton TM, Stamm WE, Humphrey PA, Hultgren SJ. 2007. Detection of intracellular bacterial communities in human urinary tract infection. PLoS Med 4:e329. doi:10.1371/journal.pmed.0040329 PMC2140087 18092884

[19] Connell I, Agace W, Klemm P, Schembri M, Mărild S, Svanborg C. 1996. Type 1 fimbrial expression enhances *Escherichia coli* virulence for the urinary tract. Proc Natl Acad Sci USA 93:9827–9832. doi:10.1073/pnas.93.18.9827 PMC38514 8790416

[20] King JE, Aal Owaif HA, Jia J, Roberts IS. 2015. Phenotypic heterogeneity in expression of the K1 polysaccharide capsule of uropathogenic *Escherichia coli* and downregulation of the capsule genes during growth in urine. Infect Immun 83:2605–2613. doi:10.1128/IAI.00188-15 PMC4468546 25870229

[21] Rosen DA, Pinkner JS, Jones JM, Walker JN, Clegg S, Hultgren SJ. 2008. Utilization of an intracellular bacterial community pathway in *Klebsiella pneumoniae* urinary tract infection and the effects of FimK on type 1 pilus expression. Infect Immun 76:3337–3345. doi:10.1128/IAI.00090-08 PMC2446714 18411285

[22] Rosen DA, Pinkner JS, Walker JN, Elam JS, Jones JM, Hultgren SJ. 2008. Molecular variations in *Klebsiella pneumoniae* and *Escherichia coli* FimH affect function and pathogenesis in the urinary tract. Infect Immun 76:3346–3356. doi:10.1128/IAI.00340-08 PMC2446687 18474655

[23] Lopatto EDB, Pinkner JS, Sanick DA, Potter RF, Liu LX, Bazán Villicaña J, Tamadonfar KO, Ye Y, Zimmerman MI, Gualberto NC, Dodson KW, Janetka JW, Hunstad DA, Hultgren SJ. 2024. Conformational ensembles in *Klebsiella pneumoniae* FimH impact uropathogenesis. Proc Natl Acad Sci USA 121:e2409655121. doi:10.1073/pnas.2409655121 PMC11441496 39288182

[24] Khater F, Balestrino D, Charbonnel N, Dufayard JF, Brisse S, Forestier C. 2015. *In silico* analysis of usher encoding genes in *Klebsiella pneumoniae* and characterization of their role in adhesion and colonization. PLoS One 10:e0116215. doi:10.1371/journal.pone.0116215 PMC4353729 25751658

[25] Stahlhut SG, Chattopadhyay S, Struve C, Weissman SJ, Aprikian P, Libby SJ, Fang FC, Krogfelt KA, Sokurenko EV. 2009. Population variability of the FimH type 1 fimbrial adhesin in *Klebsiella pneumoniae*. J Bacteriol 191:1941–1950. doi:10.1128/JB.00601-08 PMC2648365 19151141

[26] Nassif X, Fournier JM, Arondel J, Sansonetti PJ. 1989. Mucoid phenotype of *Klebsiella pneumoniae* is a plasmid-encoded virulence factor. Infect Immun 57:546–552. doi:10.1128/iai.57.2.546-552.1989 PMC313131 2643575

[27] Bengoechea JA, Sa Pessoa J. 2019. *Klebsiella pneumoniae* infection biology: living to counteract host defences. FEMS Microbiol Rev 43:123–144. doi:10.1093/femsre/fuy043 PMC6435446 30452654

[28] Tarkkanen AM, Allen BL, Williams PH, Kauppi M, Haahtela K, Siitonen A, Orskov I, Orskov F, Clegg S, Korhonen TK. 1992. Fimbriation, capsulation, and iron-scavenging systems of *Klebsiella* strains associated with human urinary tract infection. Infect Immun 60:1187–1192. doi:10.1128/iai.60.3.1187-1192.1992 PMC257611 1347287

[29] Ovchinnikova OG, Treat LP, Teelucksingh T, Clarke BR, Miner TA, Whitfield C, Walker KA, Miller VL. 2023. Hypermucoviscosity regulator RmpD interacts with Wzc and controls capsular polysaccharide chain length. mBio 14:e00800-23. doi:10.1128/mbio.00800-23 PMC10294653 37140436

[30] Khadka S, Ring BE, Walker RS, Krzeminski LR, Pariseau DA, Hathaway M, Mobley HLT, Mike LA. 2023. Urine-mediated suppression of *Klebsiella pneumoniae* mucoidy is counteracted by spontaneous Wzc variants altering capsule chain length. mSphere 8:e00288-23. doi:10.1128/msphere.00288-23 PMC10597399 37610214

[31] Ernst CM, Braxton JR, Rodriguez-Osorio CA, Zagieboylo AP, Li L, Pironti A, Manson AL, Nair AV, Benson M, Cummins K, Clatworthy AE, Earl AM, Cosimi LA, Hung DT. 2020. Adaptive evolution of virulence and persistence in carbapenem-resistant *Klebsiella pneumoniae*. Nat Med 26:705–711. doi:10.1038/s41591-020-0825-4 PMC9194776 32284589

[32] Martin RM, Cao J, Brisse S, Passet V, Wu W, Zhao L, Malani PN, Rao K, Bachman MA. 2016. Molecular epidemiology of colonizing and infecting isolates of *Klebsiella pneumoniae*. mSphere 1:e00261-16. doi:10.1128/mSphere.00261-16 PMC5071533 27777984

[33] Vornhagen J, Roberts EK, Unverdorben L, Mason S, Patel A, Crawford R, Holmes CL, Sun Y, Teodorescu A, Snitkin ES, Zhao L, Simner PJ, Tamma PD, Rao K, Kaye KS, Bachman MA. 2022. Combined comparative genomics and clinical modeling reveals plasmid-encoded genes are independently associated with *Klebsiella* infection. Nat Commun 13:4459. doi:10.1038/s41467-022-31990-1 PMC9343666 35915063

[34] Rao K, Patel A, Sun Y, Vornhagen J, Motyka J, Collingwood A, Teodorescu A, Baang JH, Zhao L, Kaye KS, Bachman MA. 2021. Risk factors for *Klebsiella* infections among hospitalized patients with preexisting colonization. mSphere 6. doi:10.1128/mSphere.00132-21 PMC8265626 34160237

[35] CDC. 2026. Urinary tract infection (catheter-associated urinary tract infection [CAUTI] and non-catheter-associated urinary tract infection [UTI]) events

[36] Wyres KL, Wick RR, Gorrie C, Jenney A, Follador R, Thomson NR, Holt KE. 2016. Identification of *Klebsiella* capsule synthesis loci from whole genome data. Microb Genom 2:e000102. doi:10.1099/mgen.0.000102 PMC5359410 28348840

[37] Wick RR, Heinz E, Holt KE, Wyres KL. 2018. Kaptive web: user-friendly capsule and lipopolysaccharide serotype prediction for *Klebsiella* genomes. J Clin Microbiol 56:e00197-18. doi:10.1128/JCM.00197-18 PMC5971559 29618504

[38] Lam MMC, Wick RR, Judd LM, Holt KE, Wyres KL. 2022. Kaptive 2.0: updated capsule and lipopolysaccharide locus typing for the *Klebsiella pneumoniae* species complex. Microb Genom 8:000800. doi:10.1099/mgen.0.000800 PMC9176290 35311639

[39] Walker KA, Treat LP, Sepúlveda VE, Miller VL. 2020. The small protein RmpD drives hypermucoviscosity in *Klebsiella pneumoniae*. mBio 11:e01750-20. doi:10.1128/mBio.01750-20 PMC7512549 32963003

[40] Kanehisa M, Goto S. 2000. KEGG: kyoto encyclopedia of genes and genomes. Nucleic Acids Res 28:27–30. doi:10.1093/nar/28.1.27 PMC102409 10592173

[41] Buffet A, Rocha EPC, Rendueles O. 2021. Nutrient conditions are primary drivers of bacterial capsule maintenance in *Klebsiella*. Proc Biol Sci 288:20202876. doi:10.1098/rspb.2020.2876 PMC7935059 33653142

[42] Khadka S, Ring BE, Pariseau DA, Mike LA. 2023. Characterization of *Klebsiella pneumoniae* extracellular polysaccharides. Curr Protoc 3:e937. doi:10.1002/cpz1.937 PMC10683871 38010271

[43] Kinney EL, Stark DJ, Khadka S, Tin CM, Hand TW, Bain W, Mike LA. 2026. Connections between *Klebsiella pneumoniae* bloodstream dynamics and serotype-independent capsule properties. Infect Immun 94:e00641-25. doi:10.1128/iai.00641-25 PMC12974125 41609496

[44] Salisbury SM, Miner TA, Kent LA, Lam MMC, Holt KE, Miller VL, Walker KA. 2026. The acquisition of *rmpADC* can increase virulence of classical *Klebsiella pneumoniae* in the absence of other hypervirulence-associated genes. mBio 17:e03122-25. doi:10.1128/mbio.03122-25 PMC12802315 41363447

[45] Mike LA, Stark AJ, Forsyth VS, Vornhagen J, Smith SN, Bachman MA, Mobley HLT. 2021. A systematic analysis of hypermucoviscosity and capsule reveals distinct and overlapping genes that impact *Klebsiella pneumoniae* fitness. PLoS Pathog 17:e1009376. doi:10.1371/journal.ppat.1009376 PMC7993769 33720976

[46] Wacharotayankun R, Arakawa Y, Ohta M, Tanaka K, Akashi T, Mori M, Kato N. 1993. Enhancement of extracapsular polysaccharide synthesis in Klebsiella pneumoniae by RmpA2, which shows homology to NtrC and FixJ. Infect Immun 61:3164–3174. doi:10.1128/iai.61.8.3164-3174.1993 PMC280984 8335346

[47] Le MN-T, Kayama S, Wyres KL, Yu L, Hisatsune J, Suzuki M, Yahara K, Terachi T, Sawa K, Takahashi S, Okuhara T, Kohama K, Holt KE, Mizutani T, Ohge H, Sugai M. 2022. Genomic epidemiology and temperature dependency of hypermucoviscous *Klebsiella pneumoniae* in Japan. Microb Genom 8. doi:10.1099/mgen.0.000827 PMC9465067 35622495

[48] Russo TA, Olson R, Fang C-T, Stoesser N, Miller M, MacDonald U, Hutson A, Barker JH, La Hoz RM, Johnson JR, Backer M, Bajwa R, Catanzaro AT, Crook D, de Almeda K, Fierer J, Greenberg DE, Klevay M, Patel P, Ratner A, Wang J-T, Zola J. 2018. Identification of biomarkers for differentiation of hypervirulent *Klebsiella pneumoniae* from classical *K. pneumoniae*. J Clin Microbiol 56:e00776-18. doi:10.1128/JCM.00776-18 PMC6113484 29925642

[49] Nucci A, Rocha EPC, Rendueles O. 2022. Adaptation to novel spatially-structured environments is driven by the capsule and alters virulence-associated traits. Nat Commun 13:4751. doi:10.1038/s41467-022-32504-9 PMC9376106 35963864

[50] Maheswari UB, Palvai S, Anuradha PR, Kammili N. 2013. Hemagglutination and biofilm formation as virulence markers of uropathogenic *Escherichia coli* in acute urinary tract infections and urolithiasis. Indian J Urol 29:277–281. doi:10.4103/0970-1591.120093 PMC3822341 24235787

[51] Shea AE, Frick-Cheng AE, Smith SN, Mobley HLT. 2022. Phenotypic assessment of clinical *Escherichia coli* isolates as an indicator for uropathogenic potential. mSystems 7:e0082722. doi:10.1128/msystems.00827-22 PMC9765037 36445110

[52] McCabe WR, Kaijser B, Olling S, Uwaydah M, Hanson LA. 1978. *Escherichia coli* in bacteremia: K and O antigens and serum sensitivity of strains from adults and neonates. J Infect Dis 138:33–41. doi:10.1093/infdis/138.1.33 355575

[53] Coggon CF, Jiang A, Goh KGK, Henderson IR, Schembri MA, Wells TJ. 2018. A novel method of serum resistance by *Escherichia coli* that causes urosepsis. mBio 9:e00920-18. doi:10.1128/mBio.00920-18 PMC6020292 29946047

[54] Morita Y, Ikeguchi H, Nakamura J, Hotta N, Yuzawa Y, Matsuo S. 2000. Complement activation products in the urine from proteinuric patients. J Am Soc Nephrol 11:700–707. doi:10.1681/ASN.V114700 10752529

[55] Doorduijn DJ, Rooijakkers SHM, van Schaik W, Bardoel BW. 2016. Complement resistance mechanisms of *Klebsiella pneumoniae*. Immunobiology 221:1102–1109. doi:10.1016/j.imbio.2016.06.014 27364766

[56] Short FL, Di Sario G, Reichmann NT, Kleanthous C, Parkhill J, Taylor PW. 2020. Genomic profiling reveals distinct routes to complement resistance in *Klebsiella pneumoniae*. Infect Immun 88:e00043-20. doi:10.1128/IAI.00043-20 PMC7375759 32513855

[57] Mobley HL, Green DM, Trifillis AL, Johnson DE, Chippendale GR, Lockatell CV, Jones BD, Warren JW. 1990. Pyelonephritogenic *Escherichia coli* and killing of cultured human renal proximal tubular epithelial cells: role of hemolysin in some strains. Infect Immun 58:1281–1289. doi:10.1128/iai.58.5.1281-1289.1990 PMC258621 2182540

[58] Walters MS, Lane MC, Vigil PD, Smith SN, Walk ST, Mobley HLT. 2012. Kinetics of uropathogenic *Escherichia coli* metapopulation movement during urinary tract infection. mBio 3:e00303-11. doi:10.1128/mBio.00303-11 PMC3273315 22318320

[59] Johnson DE, Lockatell CV, Russell RG, Hebel JR, Island MD, Stapleton A, Stamm WE, Warren JW. 1998. Comparison of *Escherichia coli* strains recovered from human cystitis and pyelonephritis infections in transurethrally challenged mice. Infect Immun 66:3059–3065. doi:10.1128/IAI.66.7.3059-3065.1998 PMC108313 9632566

[60] Hagan EC, Mobley HLT. 2009. Haem acquisition is facilitated by a novel receptor Hma and required by uropathogenic *Escherichia coli* for kidney infection. Mol Microbiol 71:79–91. doi:10.1111/j.1365-2958.2008.06509.x PMC2736550 19019144

[61] Martinez JJ, Mulvey MA, Schilling JD, Pinkner JS, Hultgren SJ. 2000. Type 1 pilus-mediated bacterial invasion of bladder epithelial cells. EMBO J 19:2803–2812. doi:10.1093/emboj/19.12.2803 PMC203355 10856226

[62] Dhakal BK, Mulvey MA. 2009. Uropathogenic *Escherichia coli* invades host cells via an HDAC6-modulated microtubule-dependent pathway. J Biol Chem 284:446–454. doi:10.1074/jbc.M805010200 PMC2610520 18996840

[63] Jerman UD, Višnjar T, Bratkovič IH, Resnik N, Pavlin M, Veranič P, Kreft ME. 2021. Attachment of cancer urothelial cells to the bladder epithelium occurs on uroplakin-negative cells and is mediated by desmosomal and not by classical cadherins. Int J Mol Sci 22:5565. doi:10.3390/ijms22115565 PMC8197456 34070317

[64] Wu XR, Kong XP, Pellicer A, Kreibich G, Sun TT. 2009. Uroplakins in urothelial biology, function, and disease. Kidney Int 75:1153–1165. doi:10.1038/ki.2009.73 PMC3717210 19340092

[65] Moll R, Wu XR, Lin JH, Sun TT. 1995. Uroplakins, specific membrane proteins of urothelial umbrella cells, as histological markers of metastatic transitional cell carcinomas. Am J Pathol 147:1383–1397. PMC1869506 7485401

[66] Lobban ED, Smith BA, Hall GD, Harnden P, Roberts P, Selby PJ, Trejdosiewicz LK, Southgate J. 1998. Uroplakin gene expression by normal and neoplastic human urothelium. Am J Pathol 153:1957–1967. doi:10.1016/S0002-9440(10)65709-4 PMC1866332 9846985

[67] Ogawa K, Johansson SL, Cohen SM. 1999. Immunohistochemical analysis of uroplakins, urothelial specific proteins, in ovarian brenner tumors, normal tissues, and benign and neoplastic lesions of the female genital tract. Am J Pathol 155:1047–1050. doi:10.1016/S0002-9440(10)65206-6 PMC1867018 10514386

[68] Liu X, Xu Q, Yang X, Heng H, Yang C, Yang G, Peng M, Chan E-C, Chen S. 2025. Capsular polysaccharide enables *Klebsiella pneumoniae* to evade phagocytosis by blocking host-bacteria interactions. mBio 16:e03838-24. doi:10.1128/mbio.03838-24 PMC11898582 39950808

[69] Connell H, Poulsen LK, Klemm P. 2000. Expression of type 1 and P fimbriae *in situ* and localisation of a uropathogenic *Escherichia coli* strain in the murine bladder and kidney. Int J Med Microbiol 290:587–597. doi:10.1016/S1438-4221(00)80006-5 11200540

[70] Arthur M, Johnson CE, Rubin RH, Arbeit RD, Campanelli C, Kim C, Steinbach S, Agarwal M, Wilkinson R, Goldstein R. 1989. Molecular epidemiology of adhesin and hemolysin virulence factors among uropathogenic *Escherichia coli*. Infect Immun 57:303–313. doi:10.1128/iai.57.2.303-313.1989 PMC313098 2563254

[71] Schaeffer AJ, Schwan WR, Hultgren SJ, Duncan JL. 1987. Relationship of type 1 pilus expression in *Escherichia coli* to ascending urinary tract infections in mice. Infect Immun 55:373–380. doi:10.1128/iai.55.2.373-380.1987 PMC260337 2879794

[72] Xia Y, Gally D, Forsman‐Semb K, Uhlin BE. 2000. Regulatory cross‐talk between adhesin operons in *Escherichia coli*: inhibition of type 1 fimbriae expression by the PapB protein. EMBO J 19:1450–1457. doi:10.1093/emboj/19.7.1450 PMC310214 10747013

[73] Lane MC, Mobley HLT. 2007. Role of P-fimbrial-mediated adherence in pyelonephritis and persistence of uropathogenic *Escherichia coli* (UPEC) in the mammalian kidney. Kidney Int 72:19–25. doi:10.1038/sj.ki.5002230 17396114

[74] Gunther NW IV, Lockatell V, Johnson DE, Mobley HLT. 2001. *In vivo* dynamics of type 1 fimbria regulation in uropathogenic *Escherichia coli* during experimental urinary tract infection. Infect Immun 69:2838–2846. doi:10.1128/IAI.69.5.2838-2846.2001 PMC98232 11292696

[75] van den Bosch JF, Verboom-Sohmer U, Postma P, De Graaff J, MacLaren DM. 1980. Mannose-sensitive and mannose-resistant adherence to human uroepithelial cells and urinary virulence of *Escherichia coli*. Infect Immun 29:226–233. doi:10.1128/iai.29.1.226-233.1980 PMC551100 6105132

[76] Gander RM, Thomas VL, Forland M. 1985. Mannose-resistant hemagglutination and P receptor recognition of uropathogenic *Escherichia coli* isolated from adult patients. J Infect Dis 151:508–513. doi:10.1093/infdis/151.3.508 2857751

[77] Godaly G, Bergsten G, Hang L, Fischer H, Frendéus B, Lundstedt A-C, Samuelsson M, Samuelsson P, Svanborg C. 2001. Neutrophil recruitment, chemokine receptors, and resistance to mucosal infection. J Leukoc Biol 69:899–906. doi:10.1189/jlb.69.6.899 11404374

[78] Hayes BW, Abraham SN. 2016. Innate immune responses to bladder infection. Microbiol Spectr 4. doi:10.1128/microbiolspec.UTI-0024-2016 PMC5242417 28084200

[79] Jacobson SH, Ostenson CG, Tullus K, Brauner A. 1992. Serum resistance in *Escherichia coli* strains causing acute pyelonephritis and bacteraemia. APMIS 100:147–153. doi:10.1111/j.1699-0463.1992.tb00854.x 1554489

[80] Hagberg L, Hull R, Hull S, Falkow S, Freter R, Edén CS. 1983. Contribution of adhesion to bacterial persistence in the mouse urinary tract. Infect Immun 40:265–272. doi:10.1128/iai.40.1.265-272.1983 PMC264844 6131870

[81] Hagberg L, Engberg I, Freter R, Lam J, Olling S, Svanborg Edén C. 1983. Ascending, unobstructed urinary tract infection in mice caused by pyelonephritogenic *Escherichia coli* of human origin. Infect Immun 40:273–283. doi:10.1128/iai.40.1.273-283.1983 PMC264845 6339403

[82] Hagberg L, Hull R, Hull S, McGhee JR, Michalek SM, Svanborg Edén C. 1984. Difference in susceptibility to Gram-negative urinary tract infection between C3H/HeJ and C3H/HeN mice. Infect Immun 46:839–844. doi:10.1128/iai.46.3.839-844.1984 PMC261623 6389367

[83] Johnson JR, Clabots C, Rosen H. 2006. Effect of inactivation of the global oxidative stress regulator oxyR on the colonization ability of *Escherichia coli* O1:K1:H7 in a mouse model of ascending urinary tract infection. Infect Immun 74:461–468. doi:10.1128/IAI.74.1.461-468.2006 PMC1346679 16369002

[84] Mobley HL, Jarvis KG, Elwood JP, Whittle DI, Lockatell CV, Russell RG, Johnson DE, Donnenberg MS, Warren JW. 1993. Isogenic P-fimbrial deletion mutants of pyelonephritogenic *Escherichia coli*: the role of alpha Gal(1-4) beta Gal binding in virulence of a wild-type strain. Mol Microbiol 10:143–155. doi:10.1111/j.1365-2958.1993.tb00911.x 7968511

[85] Hyun M, Lee JY, Kim HA, Ryu SY. 2019. Comparison of *Escherichia coli* and *Klebsiella pneumoniae* acute pyelonephritis in Korean patients. Infect Chemother:130–141. doi:10.3947/ic.2019.51.2.130 PMC6609746 31270992

[86] Strömberg N, Nyholm PG, Pascher I, Normark S. 1991. Saccharide orientation at the cell surface affects glycolipid receptor function. Proc Natl Acad Sci USA 88:9340–9344. doi:10.1073/pnas.88.20.9340 PMC52710 1681550

[87] Pariseau DA, Ring BE, Khadka S, Mike LA. 2024. Cultivation and Genomic DNA extraction of *Klebsiella pneumoniae*. Curr Protoc 4:e932. doi:10.1002/cpz1.932 PMC11407547 38279957

[88] Argimón S, David S, Underwood A, Abrudan M, Wheeler NE, Kekre M, Abudahab K, Yeats CA, Goater R, Taylor B, Harste H, Muddyman D, Feil EJ, Brisse S, Holt K, Donado-Godoy P, Ravikumar KL, Okeke IN, Carlos C, Aanensen DM, NIHR Global Health Research Unit on Genomic Surveillance of Antimicrobial Resistance. 2021. Rapid genomic characterization and global surveillance of *Klebsiella* using Pathogenwatch. Clin Infect Dis 73:S325–S335. doi:10.1093/cid/ciab784 PMC8634497 34850838

[89] Lam MMC, Wick RR, Watts SC, Cerdeira LT, Wyres KL, Holt KE. 2021. A genomic surveillance framework and genotyping tool for *Klebsiella pneumoniae* and its related species complex. Nat Commun 12:4188. doi:10.1038/s41467-021-24448-3 PMC8263825 34234121

[90] Ong CY, Beatson SA, Totsika M, Forestier C, McEwan AG, Schembri MA. 2010. Molecular analysis of type 3 fimbrial genes from *Escherichia coli*, *Klebsiella* and *Citrobacter* species. BMC Microbiol 10:183. doi:10.1186/1471-2180-10-183 PMC2900259 20576143

[91] Olson RD, Assaf R, Brettin T, Conrad N, Cucinell C, Davis JJ, Dempsey DM, Dickerman A, Dietrich EM, Kenyon RW, et al.. 2023. Introducing the bacterial and viral bioinformatics resource center (BV-BRC): a resource combining PATRIC, IRD and ViPR. Nucleic Acids Res 51:D678–D689. doi:10.1093/nar/gkac1003 PMC9825582 36350631

[92] Anderson MT, Mitchell LA, Zhao L, Mobley HLT. 2017. Capsule production and glucose metabolism dictate fitness during *Serratia marcescens* bacteremia. mBio 8:e00740-17. doi:10.1128/mBio.00740-17 PMC5442460 28536292

[93] Zychlinsky Scharff A, Albert ML, Ingersoll MA. 2017. Urinary tract infection in a small animal model: transurethral catheterization of male and female mice. JoVE:130. doi:10.3791/54432 PMC5755510 29286380

