# Supplementary material for: Phenotypically diverse Klebsiella pneumoniae urinary tract infection isolates consistently cause pyelonephritis
Source: Infect Immun. 2026 Oct 12;94(11):e00508-26. doi: 10.1128/iai.00508-26 (PMC13750118; doi:10.1128/iai.00508-26)
Supplement: Supplementary file 1 [file iai.00508-26-s0001.pdf]

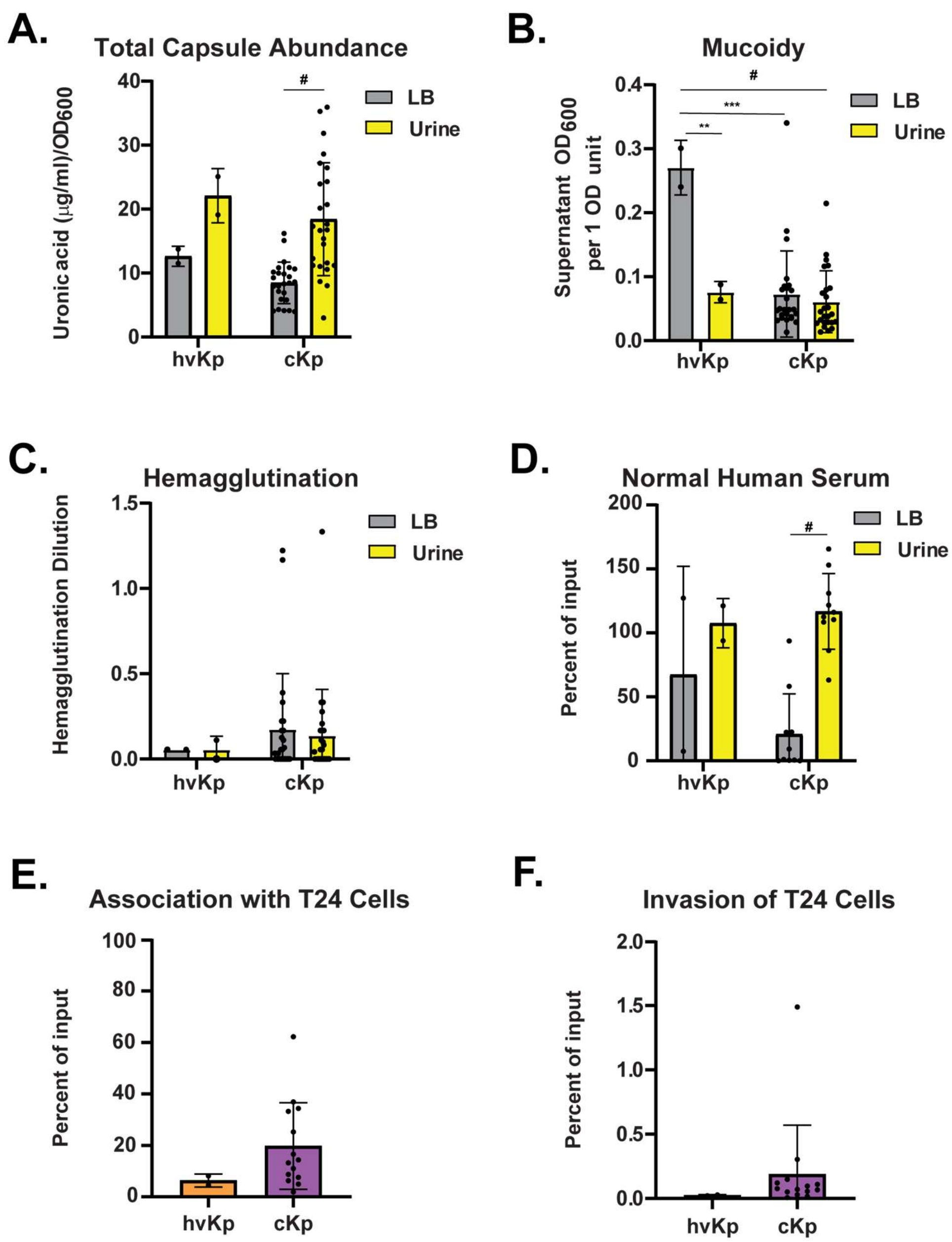

Figure S1. Phenotypic comparisons between hvKp and cKp strains.

Values from data presented in the main figures were plotted and stratified by *K. pneumoniae* pathotype. Each dot represents the mean of a single hvKp or cKp strain. Phenotypic differences between growth in sterile-filtered pooled human urine and LB were compared for (**A**) cell-associated capsule abundance, (**B**) mucoidy, (**C**) hemagglutination, and (**D**) human serum resistance. Due to limited data, urine and LB data were combined and compared between hvKp and cKp for (**E**) T24 cell association and (**F**) T24 cell invasion. Each dot represents the mean of one strain and error bars represent standard error of the mean. Statistical significance was determined using (**A-D**) one-way ANOVA with Tukey's post-test or (**E-F**) Mann-Whitney U test (* P <0.05; ** P < 0.01; *** P < 0.001; # P < 0.0001).

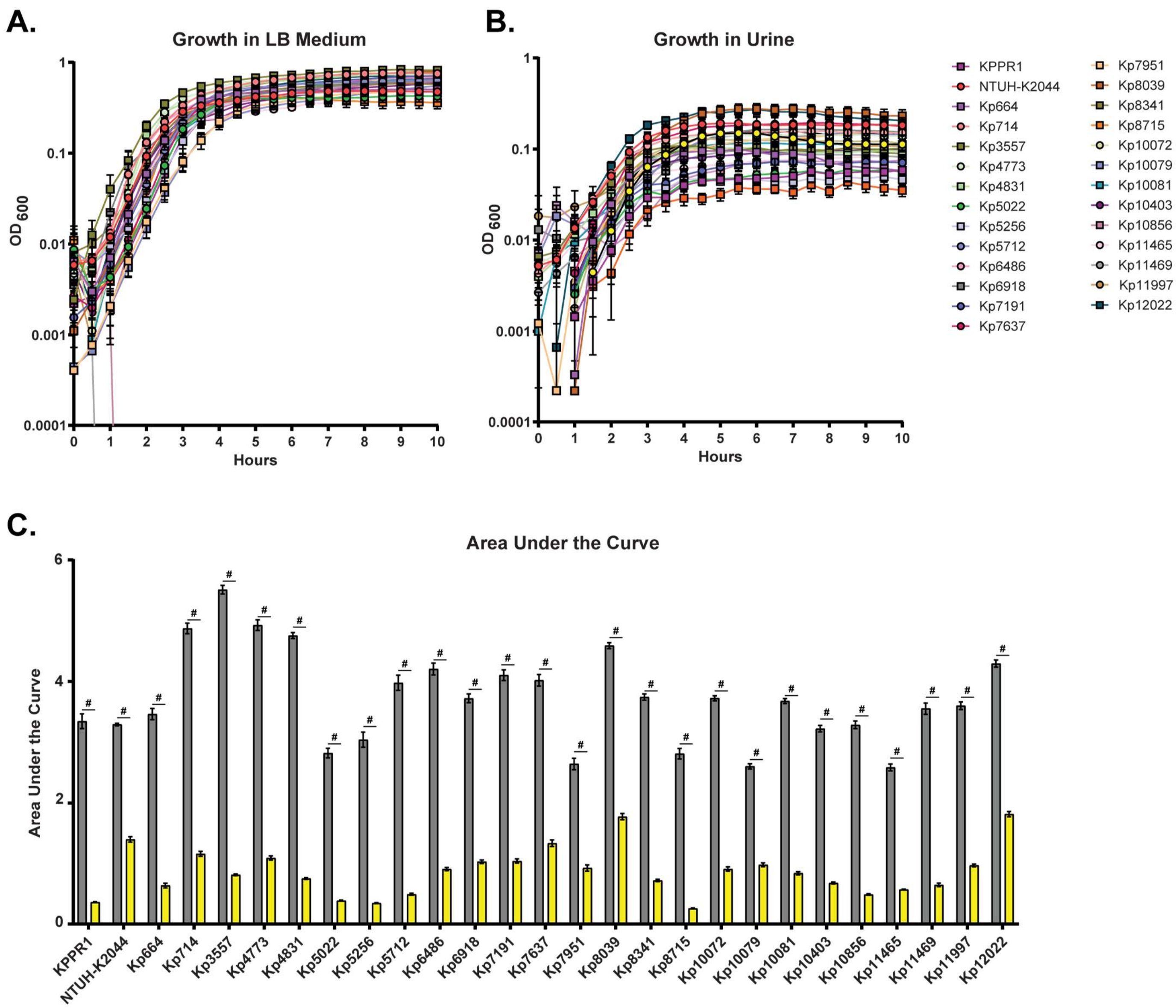

Figure S2. *K. pneumoniae* achieves a lower growth density in urine relative to LB.

All strains were cultured in (**A**) LB medium and (**B**) sterile-filtered pooled human urine and then back-diluted to an $OD6_{00}$ 0.001 in the respective medium. Cultures were incubated with shaking for 10 h at 37 º C and the density measured every 30 minutes. All growth assays were performed ≥ 3 independent times, in triplicate. (**C**) The data presented are the mean of the area under the curve and error bars represent standard error of the mean. Gray bars represent growth in LB, while yellow bars represent growth in urine. Statistical significance was determined using student's t-test (# P <0.0001).

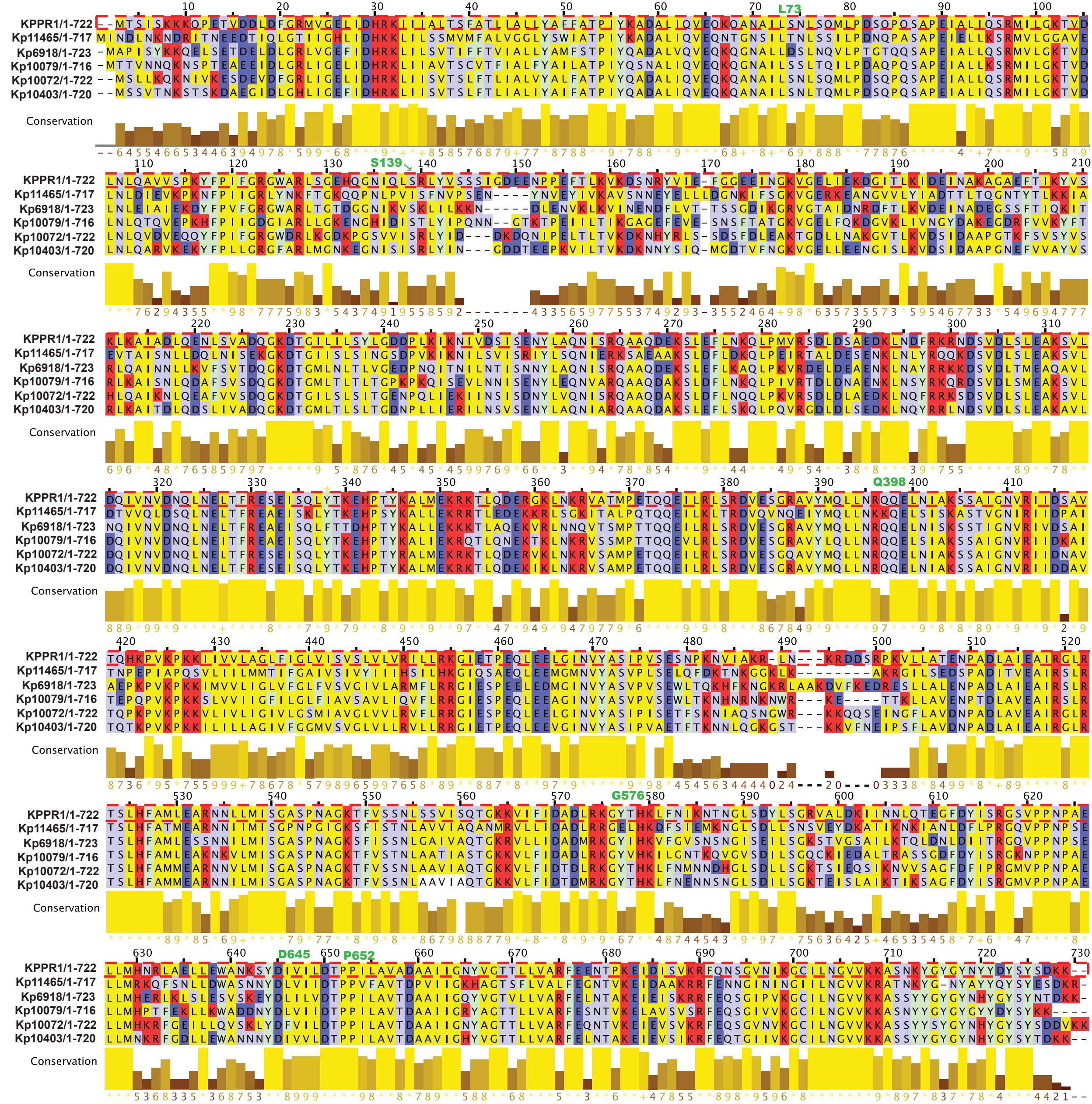
KPPR1/1-722
Kp11465/1-717
Kp6918/1-723
Kp10079/1-716
Kp10072/1-722
Kp10403/1-720
Conservation
L73
S139
Q398
G576
D645
P652

Figure S3. Wzc alignment from mucoid cKp strains.

Predicted open reading frames (ORFs) of *wzc* were obtained from unassembled genomes available in the NCBI Sequence Read Archive (SRA). An alignment of the translated ORFs was created using Clustal Omega, and the results were visualized using Jalview. The color scheme represents different amino acid residue properties: red for basic, dark blue for acidic, light blue for uncharged polar, green for aromatic, and yellow for aliphatic residues. The level of amino acid residue conservation is depicted through histograms positioned below the alignment. Residue number is relative to the start of the Kp11465 Wzc sequence. Wzc variants previously associated with mucoidy include: L73, S139, Q398, G576, D645, and P652 (notated in green text).[30, 31, 39, 49]

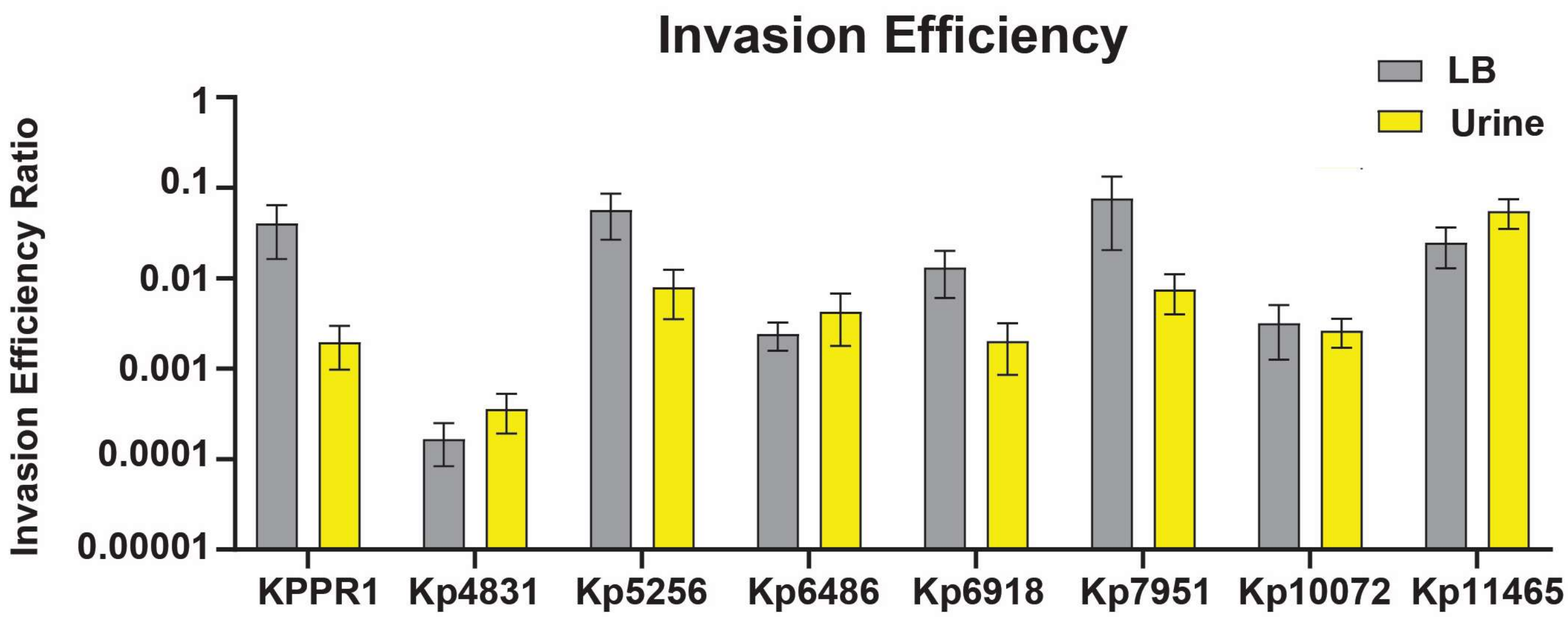

Figure S4. cKp strains do not exhibit a significant difference in invasion efficiency compared to external association.

Values from the Figure 4 raw CFU counts were used to calculate the number of intracellular bacteria relative to extracellular-associated bacteria ('invasion efficiency'). Intracellular CFUs (100 μg/mL gentamicin treatment) were subtracted from total cell-associated CFUs (without treatment) to determine extracellular-associated CFUs. Then, intracellular CFUs were divided by extracellular-associated CFUs to quantify invasion efficiency. Statistical significance was tested using two-way ANOVA with Bonferroni post-test, no significant differences were detected.

Strain	Accession Number
**KPPR1**	GCA_000742755.1
**NTUH-K2044**	GCA_000009885.1
**664**	SRS1664169
**714**	SRS1664168
**3557**	SRS12803755
**4773**	SRS12904747
**4831**	SRS12904751
**5022**	SRS12904764
**5256**	SRS12904775
**5712**	SRS12904710
**6486**	SRS12803797
**6918**	SRS12803740
**7191**	SRS12803745
**7637**	SRS12803752
**7951**	SRS12803757
**8039**	SRS12803761
**8341**	SRS12803776
**8715**	SRS12803787
**8341**	SRS12803776
**10072**	SRS12902321
**10079**	SRS12902234
**10081**	SRS12902235
**10403**	SRS12902267
**10856**	SRS12902291
**11465**	SRS12902311
**11469**	SRS12902313
**11997**	SRS12902325
**12022**	SRS12902327

Supplemental Table 1. SR accession numbers for *Klebsiella* UTI isolates. KPPR1 and NTUH-K2044 genomes were preassembled in the NCBI database. The rest of the strains were assembled using BV-BRC genome assembly.

Klebsiella strain	UPEC CFT073			UPEC UTI89			UPEC (MS2027)
	FimA (c5393)	PapA (c3592)	PapA2 (c5188)	FimA (C5011)	PapA (C4895)	SfaA (C1109)	MrkA
KPPR1	81	31	32	83	0	57	95
NTUH-K2044	81	34	31	83	0	57	94
Kp664	81	41	37	83	39	57	95
Kp714	81	40	36	83	39	57	94
Kp3557	81	34	31	83	0	57	95
Kp4773	81	40	36	83	39	60	95
Kp4831	81	40	36	83	39	57	94
Kp5022	81	34	31	83	0	57	94
Kp5256	81	34	31	83	0	57	95
Kp5712	81	34	31	83	0	57	95
Kp6486	81	33	33	82	0	65	93
Kp6918	81	34	31	83	0	57	94
Kp7191	81	34	31	83	0	57	95
Kp7637	81	40	36	83	39	57	95
Kp7951	30	0	0	32	0	34	26
Kp8039	83	34	31	83	0	60	94
Kp8341	81	34	31	83	0	64	95
Kp8715	81	34	31	83	0	64	94
Kp10072	81	34	31	83	0	60	94
Kp10079	81	35	32	83	0	65	93
Kp10081	81	33	25	82	25	65	93
Kp10403	81	34	32	83	26	63	94
Kp10856	81	34	31	83	0	57	94
Kp11465	81	40	36	83	39	57	95
Kp11469	81	34	31	83	0	60	95
Kp11997	81	34	31	83	0	57	94
Kp12022	81	34	31	83	0	57	94

Supplemental Table 2. Percent identity between uropathogenic *E. coli* and *Klebsiella* major pilin subunits. Each number indicates percent identity between the major pilin subunit sequences of the indicated genome.

Strain	LB			Urine		
	Doubling Time	Area Under the Curve	Endpoint OD	Doubling Time (h)	Area Under the Curve	Endpoint OD_{600}
KPPR1	0.55	3.35	0.683	1.46	0.38	0.058
NTUH-K2044	0.83	3.29	0.474	0.92	1.4	0.182
Kp664	0.63	3.47	0.683	1.05	0.64	0.058
Kp714	0.81	4.87	0.474	1.08	1.16	0.151
Kp3557	0.96	5.51	0.505	1.11	0.81	0.102
Kp4773	0.83	4.93	0.573	1.04	1.09	0.14
Kp4831	0.92	4.76	0.358	0.95	0.75	0.096
Kp5022	0.56	2.82	0.696	1.81	0.39	0.056
Kp5256	0.53	3.04	0.55	1.12	0.35	0.046
Kp5712	0.56	3.98	0.65	1.61	0.49	0.071
Kp6486	0.89	4.21	0.591	0.69	0.91	0.113
Kp6918	0.99	3.72	0.618	0.79	1.03	0.153
Kp7191	0.6	4.11	0.478	0.77	1.04	0.148
Kp7637	0.54	4.02	0.608	0.82	1.33	0.177
Kp7951	0.55	2.64	0.63	0.84	0.92	0.15
Kp8039	0.58	4.59	0.723	0.67	1.78	0.231
Kp8341	0.82	3.74	0.573	0.81	0.72	0.089
Kp8715	0.6	2.81	0.358	1.06	0.26	0.035
Kp10072	0.68	3.73	0.696	0.77	0.91	0.115
Kp10079	0.49	2.6	0.55	0.9	0.98	0.144
Kp10081	0.69	3.68	0.65	0.9	0.84	0.112
Kp10403	0.88	3.23	0.591	1.05	0.68	0.097
Kp10856	0.52	3.29	0.618	0.86	0.51	0.075
Kp11465	0.79	2.59	0.478	1.17	0.57	0.084
Kp11469	0.66	3.55	0.608	0.78	0.65	0.081
Kp11997	0.7	3.6	0.63	1.63	0.97	0.107
Kp12022	0.73	4.3	0.723	0.94	1.82	0.204

Supplemental Table 3. Growth characteristics of strains in LB and urine.
